# The antibacterial activity of polyoxometalates: structures, antibiotic effects and future perspectives†
†Electronic supplementary information (ESI) available: Additional MIC tables, additional compound figures and additional structure-relationship graphs. See DOI: 10.1039/c7cc07549a


**DOI:** 10.1039/c7cc07549a

**Published:** 2018-01-12

**Authors:** Aleksandar Bijelic, Manuel Aureliano, Annette Rompel

**Affiliations:** a Universität Wien , Fakultät für Chemie , Institut für Biophysikalische Chemie , Althanstraße 14 , 1090 Wien , Austria . Email: annette.rompel@univie.ac.at ; http://www.bpc.univie.ac.at; b CCMar , FCT , Faculdade de Ciências e Tecnologia , Universidade do Algarve , 8000-139 Faro , Portugal

## Abstract

Polyoxometalates (POMs) are, mostly anionic, metal oxide compounds that span a wide range of tunable physical and chemical features rendering them very interesting for biological purposes, an continuously emerging but little explored field. Due to their biological and biochemical effects, including antitumor, -viral and -bacterial properties, POMs and POM-based systems are considered as promising future metallodrugs. In this article, we focus on the antibacterial activity of POMs and their therapeutic potential in the battle against bacteria and their increasing resistance against pharmaceuticals. Recent advances in the synthesis of POMs are highlighted, with emphasis on the development and properties of biologically active POM-based hybrid and nanocomposite structures. By analysing the antibacterial activity and structure of POMs, putative mode of actions are provided, including potential targets for POM–protein interactions, and a structure–activity-relationship was established for a series of POMs against two bacteria, namely *Helicobacter pylori* and *Streptococcus pneumoniae.*

## Abstract 2

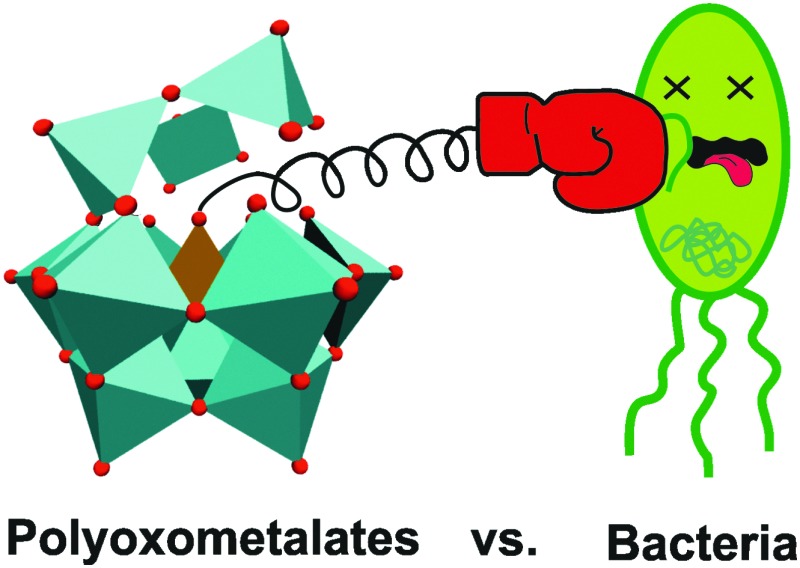
This Feature Article focuses on the antibacterial activity of POMs and POM-based hybrid and nanocomposite structures highlighting recent advances in the synthesis of biologically active POM systems and providing the state of the art in this research field.

## 1. Introduction

Polyoxometalates (POMs) are a class of discrete, mostly anionic, clusters of early transition metal oxides (mostly Mo-, W- and V-oxides) exhibiting a broad diversity of structures and outstanding properties.^1^ There are two major types of POMs, namely isopoly- and heteropolyoxoanions with the general formulas [M_*m*_O_*y*_]^*n*–^ and [X_*x*_M_*m*_O_*y*_]^*n*–^, respectively, where M represents the addenda atom, which is mostly restricted to W, Mo and V, and X the central heteroatom, which can be almost any other element. A myriad of POM structures have been synthesized in the last decades and their broad variety of physicochemical properties led to their application in various fields such as catalysis,^2^ photochemistry,^3^ electrochemistry,^4^ material science,^5^ protein crystallography^6^,^7^ and medicine.^8^ Biological applications of POMs are very promising as almost every feature that affects the interaction of POMs with biological target macromolecules can be altered in order to enhance their beneficial activities on the respective biological system.^6^,^9^ With targeted and reproducible synthetic routes, it is nowadays possible to obtain tailor-made POM systems, including organic–inorganic hybrids and other nanocomposites, with enhanced biological properties.^10^–^17^ The foundation for the research of biologically active POMs has been laid in 1970, when Chermann *et al.* discovered the inhibitory effect of silicotungstic acid on murine leukaemia and sarcoma viruses.^18^ This led to systematic studies of the antiviral effect of this and other POMs revealing that enzyme inhibition (*e.g.* inactivation of reverse transcriptase in the case of HIV) plays a major role for their activity.^19^–^21^ Further biological activities were consequently discovered: the insulin mimetic effect of POMs to combat diabetes;^22^–^25^ anticancer^23^,^26^,^27^ and antibiotic^23^,^27^,^28^ activities, and the antiparasitic potential of decavanadate against leishmania.^29^ It is believed that in many cases these POM-associated biological activities are the result of POM–protein/POM–enzyme interactions, which are of electrostatic nature as evidenced by crystallographic studies showing that the negatively charged metal clusters are mainly found within or at positively charged regions of the proteins.^6^,^30^–^36^ However, also covalent interactions between biomacromolecules and POMs are possible as was shown by our group.^35^ The inhibition of certain enzymes by POMs can trigger the impairment of vital cell functions.^37^ In this regard, the group of Aureliano extensively studied the interaction between POMs, especially polyoxovanadates (POVs), and diverse biochemical targets like ion pumps.^6^,^38^–^40^ However, the exact mode of the biological activity of POMs is still elusive and thus also the POM's target biomolecules, which are responsible for the observed biological effects. This article focuses on antibacterially active POMs as bacteria pose a threat to global public health, especially due to the rapid emergence of resistant bacteria occurring worldwide. Besides the activity of purely inorganic POMs, the antimicrobial potentials of POM-based hybrid and nanocomposite structures represent highlights of this article emphasizing the recent developments in the synthetic approach of POMs. Using our experience regarding POM–protein interactions, we tried to deduce structure–activity-relationships and potential targets to provide possible mode of actions for the antibacterial activity of POMs.

## 2. Antibacterial activity of inorganic POMs

The antibacterial activity of purely inorganic POMs can be subdivided into two major modes, namely (1) synergistic and (2) direct antibacterial activity. Most of the inorganic POMs did not exhibit significant antibacterial activity at pharmacologically acceptable concentrations but were active in synergy with conventional antibiotics.

### 2.1. Synergistic activity of inorganic POMs

**Fig. 1:**
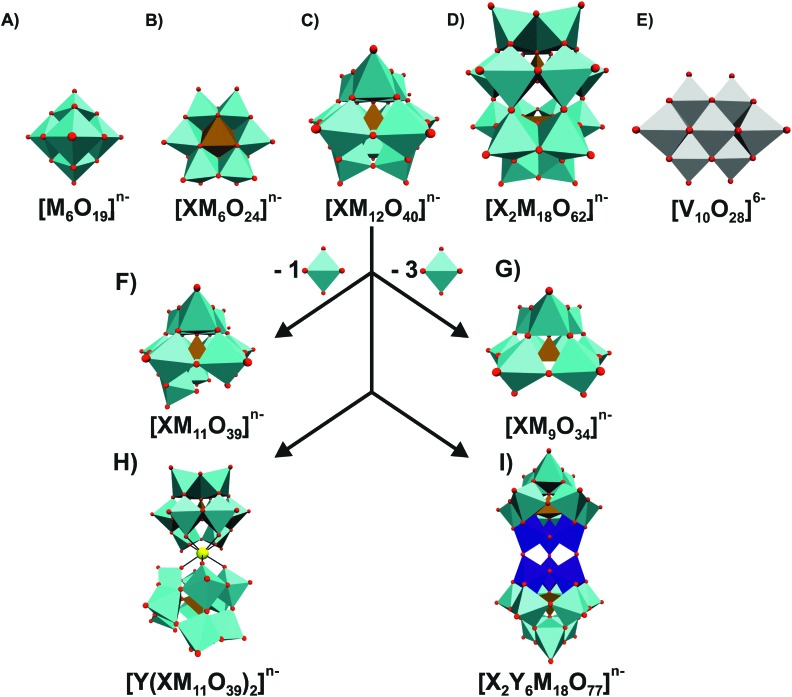
Overview of most prominent and commonly used POM structures. (A) Lindqvist, (B) Anderson-Evans, (C) Keggin, (D) Wells–Dawson, (E) decavanadate, (F) monolacunary Keggin, (G) trilacunary Keggin, (H) Keggin sandwich and (I) double Keggin structure are shown in polyhedra representation mode. X = heteroatom (ochre polyhedra), M = addenda atom (cyan polyhedra) and Y = other metal (dark blue octahedra and yellow ball, respectively). The scheme within the figure illustrates how the mono- and trilacunary Keggin structures are deduced from the fully saturated parent Keggin structure by subtracting one and three MO_6_ octahedra, respectively, and the origin of the two large Keggin-structures (H) and (I). Vanadium atoms are shown as grey octahedra, whereas oxygen atoms are illustrated as small red spheres.

As is often the case with discoveries, it was serendipity that the group of Tajima revealed the antibacterial properties of POMs in 1993.^28^ Back then, they reported on an aged mixture of tungstate and phosphate named ‘Factor T’, which greatly enhanced the antibacterial effect of β-lactam antibiotics in methicillin-resistant *Staphylococcus aureus* (MRSA) strains, which is a Gram-positive bacteria and one of the most resistant ones around the world. Years later, namely in 1997, ‘Factor T’ was identified as the lacunary Keggin POM species [PW_11_O_39_]^7–^ (Fig. 1F).^41^ [PW_11_O_39_]^7–^ showed only synergistic effects with β-lactam-based antibiotics and was only active on MRSA, *Staphylococcus epidermis* and *Staphylococcus auricularis*. Inspired by these findings, the same group investigated the antibacterial activity of β-lactam antibiotics in synergy with in total 76 POMs (Tables S1 and S2, ESI†).^42^–^46^ Fig. 1 illustrates the most prominent POM structures that were used for these antibacterial studies. The results revealed that especially Keggin- (including lacunary, double and sandwich structures) and Wells–Dawson-type (including lacunary structures) structures were most potent in terms of sensitizing the MRSA strains SR3605 (constitutive resistance) and ATCC43300 (inducible resistance) towards β-lactam antibiotics. While almost all investigated polyoxotungstates (POTs) showed promising synergistic activity by exhibiting an average fractional inhibitory concentration (FIC) index of <0.5 (average FIC_POTs_ ∼ 0.141), the tested polyoxomolybdates (POMos) and polyoxovanadates (POVs) did not exhibit any significant effect with the β-lactam antibiotic on neither MRSA strain (average FIC_POMos_ ∼ 0.725 and FIC_POVs_ ∼ 0.740).^42^–^45^ Some POMs like the Wells–Dawson-type [P_2_W_18_O_62_]^6–^ (Fig. 1D) and the Keggin-type [SiMo_12_O_40_]^4–^ (Fig. 1C) exhibited sensitizing effects also against vancomycin-resistant *Staphylococcus aureus* (VRSA) strains, which is surprising for the latter POM as POMos were in general ineffective against MRSA.^42^,^43^ Some lacunary Keggin structures (Fig. 1F and G), especially tungstates, were more effective than their fully saturated parent structure suggesting a special role for the lacuna.^44^,^45^ For example, the FIC of [SiW_12_O_40_]^4–^ against the MRSA strain SR3605 is 0.094, whereas the value for the corresponding lacunary structure [SiW_11_O_39_]^8–^ is 0.041. However, a series of lacunary-substituted [SiW_11_O_39_]^8–^ structures, where the lacuna was filled by V^IV^O, Cr^III^, Mn^II^, Fe^II/III^, Co^II^, Ni^II^, Cu^II^, La^III^, Yb^III^ or Bi^III^, had very similar or even stronger sensitizing effects than the unsubstituted species (*e.g.* FIC against MRS394-1 of [SiW_11_O_39_]^8–^ = 0.041 *vs.* FIC of [SiW_11_O_39_Co(ii)]^6–^ = 0.033) indicating that the hole in the lacunary structure has no activity-enhancing effect.^44^,^45^ Furthermore, there was no obvious correlation between the activity and the substituted metal.

It is known that the β-lactam resistance of bacteria is acquired by the expression of a peptidoglycan-synthetic enzyme that possesses a low affinity for β-lactam, namely the penicillin-binding-protein 2a (PBP2a), which is encoded by the *mecA* gene.^47^ Yamase group demonstrated that POTs ([P_2_W_18_O_62_]^6–^ and [PTi_2_W_10_O_40_]^7–^) affect the transcription process of PBP2a in MRSA.^42^,^43^,^48^ Suppression of PBP2a formation inhibits the resistance mechanism of MRSA and increases its susceptibility towards β-lactam antibiotics, which then interfere with the cell wall generation. POTs were also shown to reduce the production of β-lactamase, an antibiotic resistance-associated enzyme, which inactivates β-lactam antibiotics by hydrolyzing their β-lactam ring.^43^ Thus, POTs are able to overcome two kinds of bacterial resistance modes against β-lactam antibiotics.

The biological reduction of POMs, more precisely [P_2_W_18_O_62_]^6–^, in MRSA and VRSA cells was observed by dispersive X-ray analysis and transmission electron microscopy revealing the accumulation of POMs at the periphery of the bacterial cells.^48^

### 2.2. Direct antibiotic activity of inorganic POMs

#### 2.2.1. Direct antibiotic activity against Gram-positive bacteria

Only a few POMs (Tables S1 and S2, ESI†), which were tested against different *Staphylococcus aureus* strains, showed significant antibacterial activity on their own accord by exhibiting a minimum inhibitory concentration (MIC) of <100 μg ml^–1^ (for comparison good antibiotic drugs have MIC values ranging from 0.001 to 10 μg ml^–1^). These POMs were [Ge_2_Ti_6_W_18_O_77_]^14–^ 43 (Fig. 1I) (MIC = 50 μg ml^–1^*vs.* SR3605, 10–25 μg ml^–1^*vs.* ATCC43300), [V_10_O_28_]^6–^ 49 (Fig. 1E) (MIC = 50 μg ml^–1^*vs.* SR3605) and [Si_2_Nb_6_W_18_O_77_]^8–^ 43 (Fig. 1I) (MIC = 25 μg ml^–1^*vs.* ATC43300). Fukuda and Yamase tested the antibacterial activity of 20 purely inorganic POMs against six strains of *Streptococcus pneumoniae* (penicillin-intermediate-resistant IID553, IID554 and penicillin-resistant BS225, BS234, BS259, BS269) without the use of any additional antibiotic (Table S3, ESI†).^50^ All tested POVs showed high antibacterial activities with MIC values in the range of 4–32 μg ml^–1^ (positive control with conventional antibiotics: 2–32 μg ml^–1^), whereas the used POTs and POMos were significantly less active with MIC values of ∼128–8000 μg ml^–1^. Two of the investigated POVs, [V_4_O_12_]^4–^ and [MnV_13_O_38_]^7–^, were subsequently tested against various other bacteria (MRSA, methicillin-susceptible *Staphylococcus aureus*, coagulase-negative *staphylococci*, *Enterococcus faecalis*, *E. coli* and *Pseudomonas aeruginosa*) and did not show any significant inhibitory activity at the investigated concentrations indicating that these POVs are selectively active against *Streptococcus pneumoniae.* The study also revealed that POVs, more precisely [V_4_O_12_]^4–^ and [V_10_O_28_]^6–^, did not only inhibit the incorporation of the substrates thymidine, uridine, leucine and glucose into the cell in a non-selective manner but also induced potassium efflux from the cells indicating that the inhibitory activity of POVs against *Streptococcus pneumoniae* is partially based on their interference with the transport of substrates and ions.^50^

The group of Aureliano extensively investigated the effects of POVs on biologically relevant macromolecules such as ion pumps and therefore it is suggested that the interaction of certain POVs such as decavanadate [V_10_O_28_]^6–^ with, for example, P-type ATPases may induce the uncoupling of ATP hydrolysis and thus transforming these ion pumps into channels, which under certain circumstances can severely affect cellular ion gradients and concomitantly lead to the death of the organism.^40^,^51^ This kind of ATPase inhibition was, among other inhibition mechanisms, also suggested for palytoxin, a marine toxin, which induces K^+^ efflux upon exposition to Na^+^/K^+^-ATPase by putatively uncoupling the ion pump and inducing the formation of channels.^40^,^52^–^54^ Decavanadate was also observed to mediate cell distortions that led to concentration-depending morphological changes of the bacterial cells.^50^ Elongated bacteria cells were observed at POV concentration less than MIC, whereas at a high concentration (100 μg ml^–1^) the cells became swollen indicating cell death (bactericidal activity). Morphological changes can be ascribed, at least partially, to changes in cytoskeletal dynamics.^55^ Considering this, [V_10_O_28_]^6–^ was also reported to affect the G-actin polymerization in animals^39^ as POVs are able to interact with actin at its ATP binding site,^56^ which might also explain some POV-mediated effects in bacteria as their cytoskeleton possesses homologous elements to actin that control the bacterial shape such as MreB.^57^ MreB is present in both Gram-positive and -negative bacteria and is involved in relevant cellular functions such as coordination of cell wall formation and chromosome segregation and therefore represents a promising target.^58^

#### 2.2.2. Direct antibiotic activity against Gram-negative bacteria

As late as 2005, the first study devoted to the antibacterial activity of POMs against a Gram-negative bacterium was published (Gram-negative bacteria possess a thinner peptidoglycan layer in comparison to Gram-positive ones but instead contain an additional membrane, namely the outer membrane).^46^ Yamase group tested 13 POMs on *Helicobacter pylori* (Table 3), which is usually found in the stomach and responsible for the majority of ulcers in the stomach and small intestine. Regarding the antibacterial effect of POMs, highly negatively charged POTs such as [KAs_4_W_40_O_140_]^27–^, [KSb_9_W_21_O_86_]^18–^, Keggin-type POTs and polyoxovanadotungstates (Fig. 1) have shown promising antibacterial activity on their own accord (MICs < 100 μg ml^–1^).^46^ The former large cryptate anion, [KAs_4_W_40_O_140_]^27–^, exhibited the highest activity and was even more active than the antibiotic drug metronidazole (MTZ) against MTZ-susceptible *Helicobacter pylori* strains when comparing the MIC values in μM units (MIC_POT_ ∼ 1.4 μM *vs.* MIC_MTZ_ ∼ 2.9–23.4 μM). However, none of the most active POTs, [KAs_4_W_40_O_140_]^27–^ (MIC = 1.4 μM/16 μg ml^–1^), [KSb_9_W_21_O_86_]^18–^ (MIC = 2.3–9.0 μM/16–64 μg ml^–1^) and [SiVW_11_O_40_]^5–^ (MIC = 5.4–87.2 μM/16–256 μg ml^–1^), exhibited synergistic effects with the antibiotics MTZ, amoxicillin and clarithromycin as indicated by FIC indices of >0.5. Furthermore, it was shown that the investigated POTs are uptaken into the cells of the Gram-negative bacterium, more precisely, into the periplasmic space or the inner membrane but not into the cytoplasm, which was also shown for Gram-positive bacteria. The authors suggested that POM incorporation into Gram-negative cells is mediated by porins.^46^ As was already the case for Gram-positive bacteria like MRSA, POMos exhibited only minor activity against the Gram-negative *Helicobacter pylori* (MIC values of >256 μg ml^–1^/140–236 μM) which is most likely due to the lower chemical stability of molybdate in comparison to tungstate, demonstrating the importance of the stability of POMs for their biological activity.

Another example of directly exhibited antibacterial activity of POMs is the photocatalytic inactivation of *Escherichia coli* (Gram-negative) and *Bacillus subtilis* (Gram-positive) by the Keggin heteropolyacids H_3_PW_12_O_40_, H_3_PMo_12_O_40_ and H_4_SiW_12_O_40_.^59^ The investigated POMs showed also some antibacterial activity in the absence of UV-irradiation but to a much lesser extent than upon UV-excitation. The POM-mediated photocatalytic inactivation performances were superior to that of TiO_2_, which is a known photocatalytic disinfectant. The concentration of inactivated cells (*e.g. Escherichia coli*), after 20 minutes of UV-irradiation, was three orders of magnitude higher in the presence of 0.05 mM H_3_PMo_12_O_40_ or 0.1 mM H_4_SiW_12_O_40_ than in the presence of 12.5 mM of the control TiO_2_, whereas in the presence of 0.35 mM H_3_PW_12_O_40_ it was only one order of magnitude higher. The antibacterial activity was ascribed to the oxidant power of the excited POMs as they were able to carry out the *in situ* alcohol dehydrogenation of methanol demonstrating their ability to exhibit damaging oxidation reactions within the cell, which, finally, could lead to cell death.

Table 1 summarizes the effects of the above described inorganic POM-types on all tested bacteria.

**Table 1:** Overview of the effect of purely inorganic POMs on all tested bacteria

POM-type	Synergistic effect on^*a*^	Ref.	No synergistic effect on^*a*^	Ref.	Antibacterially active on (alone)	Ref.	Antibacterially inactive on (alone)	Ref.
Polyoxotungstates:	MR/VR *S. aureus*^(+)^	42–46	MR *S. haemolyticus*^(+)^	45 and 60	*S. pneumoniae* ^(+)^	50	MR/VR *S. aureus*^(+)^	42–46
	MR *S. epidermis*^(+)^	60	MR *S. capitis*^(+)^	45 and 60	(very low activity)		MR *S. epidermis*^(+)^	43 and 60
	MR *S. auricularis*^(+)^	60	MR *S. caprae*^(+)^	45 and 60	*B. subtilis* ^(+)^ ^*b*^	59	MR *S. auricularis*^(+)^	60
	MS *S. haemolyticus*^(+)^	60	MR *S. saprolyticus*^(+)^	45 and 60	DS *H. pylori*^(–)^^*c*^	46	MS *S. haemolyticus*^(+)^	45 and 60
	MZR *H. pylori*^(–)^	46	MR *S. sciuri*^(+)^	45 and 60	MZR *H. pylori*^(–)^^*c*^	46	MR *S. haemolyticus*^(+)^	45 and 60
	CLR *H. pylori*^(–)^	46	PR *S. pneumoniae*^(+)^	50	CLR *H. pylori*^(–)^^*c*^	46	MR *S. capitis*^(+)^	60
			PR *E. faecalis*^(+)^	60	*E. coli* ^(–)^ ^*b*^	59	MR *S. caprae*^(+)^	60
			PR *E. coli*^(–)^	60			MR *S. saprolyticus*^(+)^	60
			PR *E. cloacae*^(–)^	60			MR *S. sciuri*^(+)^	60
			PR *P. aeruginosa*^(–)^	60			PR *S. pneumoniae*^(+)^	60
			BLR *S. marcescens*^(–)^	60			PR *E. faecalis*^(+)^	60
			CPR *K. pneumoniae*^(–)^	60			PR *E. coli*^(–)^	60
							PR *E. cloacae*^(–)^	60
							PR *P. aeruginosa*^(–)^	60
							BLR *S. marcescens*^(–)^	60
							CPR *K. pneumoniae*^(–)^	60

Polyoxomolybdates:	MR/VR *S. aureus*^(+)^ (only [SiMo_12_O_40_]^4–^)	42–46	MR/VR *S. aureus*^(+)^ (except [SiMo_12_O_40_]^4–^)	42–46	*S. pneumoniae* ^(+)^ (very low activity)	50	MR/VR *S. aureus*^(+)^	42–46
					DS *H. pylori*^(–)^ (very low activity)	46		
					MZR *H. pylori*^(–)^ (very low activity)	46		
					CLR *H. pylori*^(–)^ (very low activity)	46		

Polyoxovanadates:	MR/VR *S. aureus*^(+)^	42, 43 and 50	MR/VR *S. aureus*^(+)^	42–46	PR *S. pneumoniae*^(+)^	50	MR/VR *S. aureus*^(+)^ (except [V_10_O_28_]^6–^)	42, 43 and 50
					MR *S. aureus*^(+)^ (only [V_10_O_28_]^6–^)	49	CoN *Staphylococci*^*(+)*^	50
							PR *E. faecalis*^(+)^	50
							*E. coli* ^(–)^	50
							*P. aeruginosa* ^(–)^	60

^*a*^POM used in combination with an antibiotic drug (β-lactam antibiotics).

^*b*^Antibacterial activity of POTs against *E. coli* and *B. subtilis via* photocatalytic inactivation (see text).

^*c*^Only Keggin and huge and highly charged POTs ([KAs_4_W_40_O_140_]^27–^ and [KSb_9_W_21_O_86_]^18–^, see text) were antibacterially active against *H. pylori* strains. ^(+)/(–)^ indicate Gram-positive and -negative bacteria, respectively. MR = methicillin resistant, VR = vancomycin-resistant, MZR = metronidazole-resistant, CLR = clarithromycin-resistant, PR = penicillin-resistant, BLR = β-lactam-resistant (in general), CPR = carbapenem-resistant, DS = drug-susceptible, CoN = coagulase negative. Note that for bacteria lacking a resistance label there was either no further information in the respective publication or a normal drug-susceptible bacterium was used. For more information regarding the exact POM-type, please see Table 3 and Tables S1–S4 (ESI).

## 3. Antibacterial activity of POM-based organic–inorganic hybrids

A major focus in the field of POMs lies on the synthesis of organic–inorganic hybrid structures, that is, the attachment of organic moieties to the POM core. This expands the already rich structural and functional versatility of POMs and opens the door to further applications.^10^,^13^,^16^ Organic functionalities are especially interesting for bioactive POMs as they do not only enhance the stability of the POMs in certain media but also their interaction with biologically important targets. In this way, the antibacterial properties of some POMs were drastically improved.

### 3.1. Organoantimony(iii)-containing POTs

**Fig. 2:**
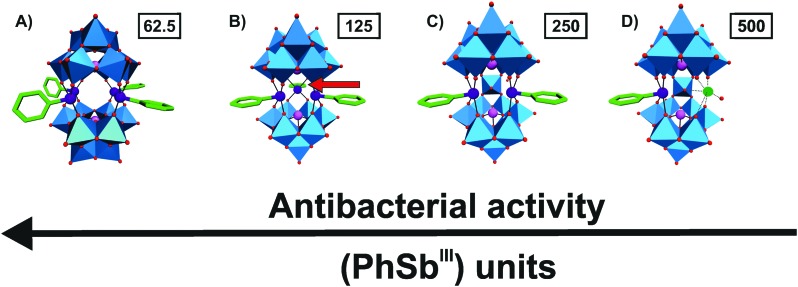
Structure–function-relationship of organoantimony(iii)-containing POTs. (A) [(PhSb^III^)_4_(A-α-As^V^W_9_O_34_)_2_],^10^–^61^ (B) [(PhSb^III^)_3_(B-α-As^III^W_9_O_33_)_2_]^12–^,^62^ (C) [(PhSb^III^)_2_As^III^_2_W_19_O_67_(H_2_O)]^10–^ 62 and (D) [(PhSb^III^){Na(H_2_O)}As^III^_2_W_19_O_67_(H_2_O)]^11–^ 62 are illustrated in the polyhedra representation mode, whereas the organic moiety is shown as sticks. The figure shows that with increasing number of attached (PhSb^III^) units the antibacterial activity of the hybrid is increased, which is indicated by the MIC values in μg ml^–1^ (numbers in small boxes) against *Escherichia coli*. The red arrow in (B) indicates the location of the third (PhSb^III^) unit as it points toward the background. Color code: tungsten, blue polyhedra; antimony, deep purple; arsenic, magenta; sodium, green (sphere); carbon, green (sticks) and oxygen, red.

The groups around Kortz and Ullrich synthesized a series of organoantimony(iii)-containing POTs and tested their antibacterial activity on a variety of bacterial strains. The first three reported organic–inorganic POM hybrids of this series were [(PhSb^III^)_4_(A-α-Ge^IV^W_9_O_34_)_2_]^12–^, [(PhSb^III^)_4_(A-α-P^V^W_9_O_34_)_2_]^10–^ and [{2-(Me_2_NCH_2_C_6_H_4_)Sb^III^}_3_(B-α-As^III^W_9_O_33_)]^3–^ (Ph = phenyl group, Me = methyl group).^61^ All three hybrid POTs were stable in aqueous media at physiological pH and showed promising antibacterial activity against both *Escherichia coli* and *Bacillus subtilis* (MIC values ranging from 40–130 μg ml^–1^) with the latter Gram-positive bacterium being slightly more sensitive to the POTs. [{2-(Me_2_NCH_2_C_6_H_4_)Sb^III^}_3_(B-α-As^III^W_9_O_33_)]^3–^ (MIC = 130 μg ml^–1^ against *E. coli* and 60 μg ml^–1^ against *B. subtilis*) was slightly less active than the other two compounds, whereas [(PhSb^III^)_4_(A-α-Ge^IV^W_9_O_34_)_2_]^12–^ (MIC = 80 and 40–80 μg ml^–1^) was more active than [(PhSb^III^)_4_(A-α-P^V^W_9_O_34_)_2_]^10–^ (MIC = 110 and 50 μg ml^–1^) indicating the importance of both the structure (bulkiness of the organic moiety) and the negative charge for the biological activity (Table S4, ESI†). In 2015, the same groups synthesized and investigated three further organoantimony(iii)-based POTs, namely the tungstoarsenates [(PhSb^III^){Na(H_2_O)}As^III^_2_W_19_O_67_(H_2_O)]^11–^, [(PhSb^III^)_2_As^III^_2_W_19_O_67_(H_2_O)]^10–^ and [(PhSb^III^)_3_(B-α-As^III^W_9_O_33_)_2_]^12–^.^62^ These isostructural compounds differed in the number of the incorporated {PhSb^III^} group. The compounds were tested on six different bacterial strains (three Gram-positive and three Gram-negative) and exhibited activity against all of them (MIC values of 7.8–500 μg ml^–1^) (Table S4, ESI†). With increasing number of attached {PhSb^III^} groups the antibacterial activity of the POT was also enhanced revealing the importance of this organic group for the biological effectiveness (Fig. 2). This was the first unambiguous structure–activity relationship for antibacterially active POMs showing the possibility to fine-tune or synthesize tailor-made hybrid POMs with enhanced bioactivity. The activity dependency on the organic moiety {PhSb^III^} was later confirmed when the hybrid compound [(PhSb^III^)_4_(A-α-As^V^W_9_O_34_)_2_]^10–^ was compared with [(OHSb^III^)_4_(A-α-As^V^W_9_O_34_)_2_]^10–^, which bears hydroxyl groups in the structure instead of phenyl groups.^63^ Antibacterial tests revealed that the hybrid POT containing the organic {PhSb^III^} groups was 4–7 times more active than its inorganic counterpart proving that the organic group is responsible for the improved antibacterial activity (MIC value range of 7.8–62.5 *vs.* 125–1000 μg ml^–1^).^61^–^63^

Another related study was testing the influence of changes within the organic group of the hybrid POTs on their antibacterial activity.^64^ For this reason, three organic–inorganic hybrid structures were synthesized similar to those mentioned before but with Me_2_NCH_2_-derivatized {PhSb^III^} groups. These hybrids were significantly less active than their non-derivatized counterparts (MIC values of 250–1000 μg ml^–1^). Even slight changes within the organic {PhSb^III^} group had led to dramatic changes in the bioactivity of the hybrid POT suggesting that both the number and kind of the organic group is a crucial key factor for the bioactivity (addition of a Me_2_HN^+^CH_2_ group to the {PhSb^III^} unit doubles the MIC value). The hybridization enhanced the stability of the POTs (at physiological conditions) and their antibacterial activity due to the insertion of hydrophobic properties into the POT core, which might assist the POT in interfering with the peptidoglycan production of the bacterial cells.

### 3.2. Quinolone-based antibiotic–POM hybrids

**Fig. 3:**
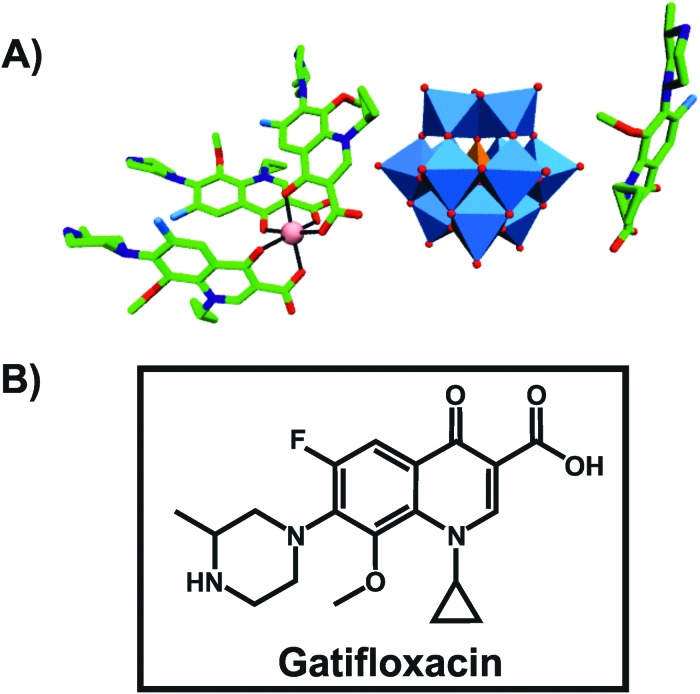
Structure of [Co^II^(C_19_FH_22_N_3_O_4_)_3_][C_19_FH_23_N_3_O_4_][HSiW_12_O_40_].^65^ (A) Structure of the organic–inorganic hybrid is shown, with the POM [HSiW_12_O_40_]^3–^ being represented as polyhedra and the organic drug as ball and stick. Color code: tungsten, blue polyhedra; silicon, orange tetrahedron; cobalt, pink (sphere connecting three gatifloxacin molecules); carbon, green; nitrogen, dark blue; fluorine, light blue; oxygen, red. (B) Structural formula of gatifloxacin is depicted to provide a better understanding of (A).

An organic–inorganic antibacterial hybrid complex consisting of the Keggin POT [HSiW_12_O_40_]^3–^, cobalt (II) cation and the clinically approved antibacterial agent gatifloxacin (C_19_FH_22_N_3_O_4_), [Co^II^(C_19_FH_22_N_3_O_4_)_3_][C_19_FH_23_N_3_O_4_][HSiW_12_O_40_] (Fig. 3), showed activity against *Staphylococcus aureus* and *Escherichia coli* (MIC = 2.52 and 2.42 μg ml^–1^, respectively).^65^ This unusual complex was slightly less active than free gatifloxacin against *Escherichia coli* when applied at the same mass concentration (MIC_Hybrid-POT_ = 2.42 μg ml^–1^*vs.* MIC_gatifloxacin_ = 1.28 μg ml^–1^). However, when used at the same molar concentration, the hybrid-complex showed the highest antibacterial activity in this study indicating synergy between the drug and [HSiW_12_O_40_]^3–^ (MIC_Hybrid-POT_ = 0.726 μg ml^–1^). The pristine [HSiW_12_O_40_]^3–^ alone was not active at all.

**Fig. 4:**
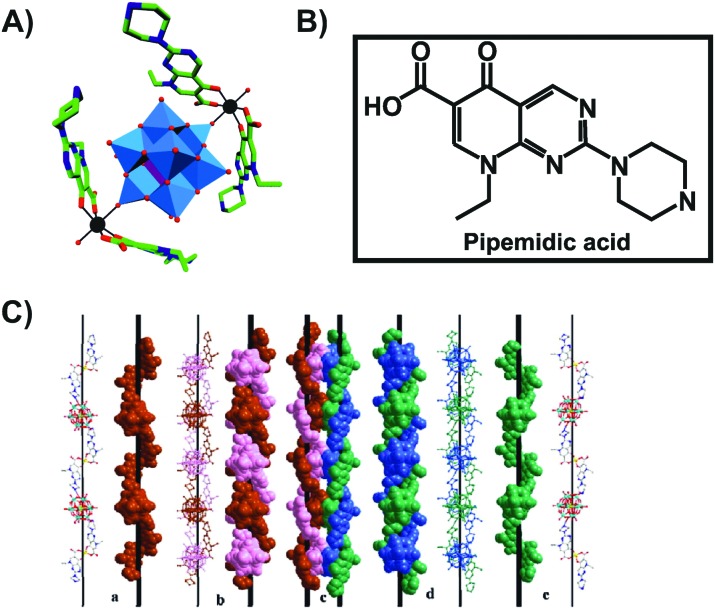
Structure of {[Zn(HPPA)_2_H_2_O]_2_[H_2_ZnW_12_O_40_]}.^66^ (A) Structure of the organic–inorganic hybrid is shown, with the POM [H_2_ZnW_12_O_40_]^4–^ being represented as polyhedra and the organic drug as ball and stick. Color code: tungsten, blue polyhedra; zinc as a heteroatom, purple tetrahedron; zinc as a linker, black; carbon, green; nitrogen, dark blue; oxygen, red. (B) Structural formula of pipemidic acid is depicted to provide a better understanding of (A). (C) The left- (a) and right- (e) hand helical chains, the left- (b) and right- (d) double-stranded helical chain and the final quadruple-stranded helices (c) are shown in both ball and stick and space-filling representation. (C) Is reprinted from Li *et al.* (2014)^66^ with permission from the copyright holder (Elsevier).

Another bioactive organic–inorganic hybrid structure consisting of a POM and a quinolone-based antibiotic is {[Zn(HPPA)_2_H_2_O]_2_[H_2_ZnW_12_O_40_]} (HPPA = pipemidic acid).^66^ This POM–drug hybrid exhibits a quadruple-stranded helical structure (Fig. 4). Its antibacterial activity against *Staphylococcus aureus* and *Escherichia coli* (judged by the size of the inhibition zone on agar plates) is similar to those of the sole antibiotic HPPA and therefore the activity might be attributed to HPPA due to the lack of unambiguous synergy.

The hybrid compound (C_9_H_8_N)_3_[NbW_5_O_19_] showed bio-activity against *Escherichia coli*, *Salmonella typhimurium* (Gram-negative), *Streptococcus agalactiae* (Gram-positive) and *Lactobacillus acidophilus* (Gram-positive) according to the size of the inhibition zones of bacterial growth.^67^ The complex is based on the Nb-substituted Lindqvist POT [NbW_5_O_19_]^3–^, which is coordinated to three quinolinium cations *via* hydrogen bonds and stabilized by π–π stacking interactions (Fig. S1, ESI†). Besides the quinoline, of which derivatives are known to be antimicrobially active, the Nb-substituted POT itself exhibited also good antibacterial activity but much lower than that of the hybrid structure. Thus, the synergistic effect of both compounds led to the high antibacterial activity of the hybrid complex, which was suggested to be based on the electrostatic/Coulombic interactions between charged ions (*e.g.* quinolinium cations) and the charged bacterial cell wall and on the presence of both proton donor and acceptor groups within the hybrid facilitating the reactions with the cell membrane and/or bacterial compounds.

## 4. Antibacterial activity of POM-based nanocomposites

Hybrid nanocomposites are of great interest for the pharmacological and medicinal fields as nanocomposite-based drug delivery systems are very promising for targeted therapies due to the possibility to deliver therapeutic agents with improved properties to their destination while minimizing adverse effects.^68^,^69^ Since the constituents of nanocomposites are usually very different in their structural, physical and chemical properties, their combination and synergy offers an extraordinary versatility in their function and bioactivity. The associated nanosize-effect increases the surface of the reactive and/or bioactive compound and thus enlarges the interaction area with the target.^70^ Furthermore, the embedding of POMs into nanocomposites reduces their toxicity.^44^ POMs have been shown to be ideal building blocks for nanocomposites due to their unique range of properties and their ability to form clusters of different sizes ranging from several angstroms up to the nanometer scale.

### 4.1. Chitosan–POM nanocomposites

**Fig. 5:**
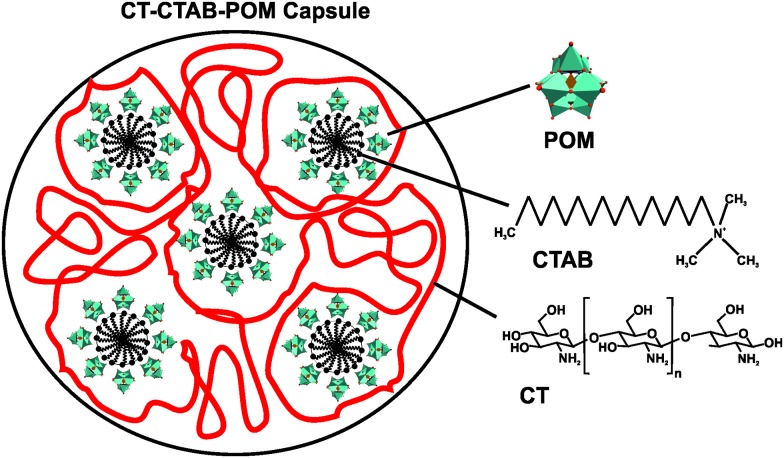
Scheme of the chitosan–cetyltrimethylammonium bromide–POM capsule.^77^ POM = [PW_12_O_40_]^3–^ or [PMo_12_O_40_]^3–^, CTAB = cetyltrimethylammonium bromide and CT = chitosan.

One well-studied nanocomposite system is that of POM and chitosan (CT).^71^ CT is a linear polysaccharide, which results from the partial *N*-deacetylation of the biopolymer chitin, and consists of randomly distributed β-(1-4)-linked d-glucosamine and *N*-acetyl-d-glucosamine. In 1992, Draget *et al.* reported on the first POM–CT gels, where CT was crosslinked with Mo(vi) polyoxyanions, however, the exact POMo species was not determined as the gel was *in situ* formed by addition of solid MoO_3_.^72^ Later studies revealed that POM-loaded chitosan dramatically enhances the uptake of POMs into the target cells. For example, the deposition of [PTi_2_W_10_O_40_]^7–^ in the human cell lines FaDu (squamous carcinoma) and HT-29 (colon adenocarcinoma) was increased by up to 16- and 24-fold (according to the amount of tungsten in the cell), respectively, *via* chitosan-loading and the cytotoxicity was remarkably reduced.^73^ In 2006, Chen *et al.* showed that a CT-Ca_3_V_10_O_28_ complex exhibited strong antibacterial activity against both Gram-negative and -positive bacteria (MIC value of 12.5 μg ml^–1^ for both *Escherichia coli* and *Staphylococcus aureus*).^49^ The CT-Ca_3_V_10_O_28_ exhibited antibacterial activity was comparable to that of the antibiotic potassium G penicillin (MIC = 12.5 μg ml^–1^) and even higher than that of ofloxacin (MIC = 25 μg ml^–1^). Both CT and [V_10_O_28_]^6–^ showed also antibacterial performance on their own accord which, however, was much lower than that of the nanocomposite (MIC_CT_ = 500 μg ml^–1^ and MIC_POV_ = 50 μg ml^–1^) (Table S4, ESI†). The antibacterial activity of CT is well known and two mechanisms have been suggested as the cause of their inhibitory effect:^74^ (1) CT as a polycation interacts with the negatively charged bacterial cell wall and in this way electrostatically disrupts the cell envelope and leads to the leakage of intracellular substances. (2) Low-molecular-weight CT is able to penetrate into the cell and inhibits the transcription of RNA from DNA by directly interacting with DNA molecules. However, the mechanism behind the antibacterial activity of both decavanadate [V_10_O_28_]^6–^ and the nanocomposite is not known but it is considered, as already mentioned earlier (Section 2.2.1), to be based on the inhibition of ion pumps (*e.g.* Na^+^/K^+^ ATPase).^40^ Also multilayer films consisting of the Keggin-type structures, [SiW_12_O_40_]^4–^ and [PMo_12_O_40_]^3–^, and CT have been prepared and showed antibacterial activity against *Escherichia coli*.^75^ Further CT–POM nanocomposites like that between CT and the redox-active [PMo_12_O_40_]^3–^ (active against *Escherichia coli*) confirmed the antibacterial potential of this complex group. Fiorani *et al.* tested several nanocomposites derived from low-molecular weight CTs and different POM-types, namely decavanadate [V_10_O_28_]^6–^, the phosphovanadomolybdic Keggin-structure [PMo_10_V_2_O_40_]^5–^ and decatungstate [W_10_O_32_]^4–^, regarding their antibacterial activity against *Escherichia coli* and their surface charge.^76^ All nanocomposites were antibacterially active in a dose-dependent manner, whereby systems exhibiting an in total more positively charge (CT-[PMo_10_V_2_O_40_]^5–^ and CT-[W_10_O_32_]^4–^) were more active than less positively charged systems (CT-[V_10_O_28_]^6–^). This suggests, in this case, a direct correlation between the activity and the ζ-potential (surface charge) of the nanocomposites as those bearing a more positive charge were able to completely inhibit the bacterial growth (at a concentration of 0.6 mg ml^–1^) and induce morphological changes of the cells. Furthermore, the activity of CT-based complexes depends on a series of factors like average molecular weight of CT, type of the crosslinking agent, average nanocomposite size and as already stated the surface charge of the nanoparticle. Another CT–POM nanocomposite study explored the difference in the antibacterial activity between a POT ([PW_12_O_40_]^3–^) and a POMo ([PMo_12_O_40_]^3–^) based spherical nanohybrid capsule.^77^ In contrast to the aforementioned CT–POM nanocomposites, where the POM was added to a pre-processed CT-containing solution, these structures were synthesized by a micelle-based approach as the surfactant cetyltrimethylammonium bromide (CTAB) was used as a cationic nucleating agent leading to CT–CTAB–POM capsules (Fig. 5). The POMo-containing capsule showed significant antibacterial activity against *Escherichia coli* according to the resazurin viability assay,^78^ (cell viability was reduced by ∼85% with 50 μg ml^–1^ of the POMo-capsule), whereas the corresponding POT-containing structure showed no activity. The reason for this discrepancy was attributed to the different shape of the nanocomposites as the POMo-based capsules were more spherical and tended to form capsules of smaller size (100–200 nm) in comparison to the POT-based systems (200–300 nm). The smaller size and the more spherical shape might facilitate the internalization and/or direct contact of the CT–CTAB–POMo capsules with the bacterial cell (wall).

### 4.2. POM-based silver and gold nanoparticles

For millennia, ionic silver (Ag^+^) has been used as a disinfectant due to its antimicrobial properties. However, it was not until the turn of the millennium that comprehensive research on the antibacterial action of silver nanoparticles (AgNPs) was done revealing their promising activity against a broad range of microbes with little systemic toxicity towards human and mammalian cells.^79^ Silver is believed to electrostatically interact with the bacterial membrane, which results in the destabilization and/or destruction of the cell wall and interference with the bacterial respiratory chain.^80^ Therefore, the production of such AgNPs in conjunction with POMs were considered promising as both compounds are bioactive and could lead to an enhanced activity. Indeed, multifunctional films consisting of [BW_12_O_40_]^5–^ and AgNPs exhibited antibacterial activity against *Escherichia coli* as agar plates incubated with the bacterium and the film did not produce bacterial colonies.^81^ On the other hand, the POT alone showed hardly any antibacterial activity indicating the necessity of the AgNPs within the film. Antibacterial tests of AgNPs, which were surface-modified by the Keggin-type POMs H_3_PW_12_O_40_ and H_3_PMo_12_O_40_, showed enhanced physical damage to both Gram-negative (*Escherichia coli*) and -positive (*Staphylococcus albus*) cells compared to the unmodified AgNPs.^82^ At a fixed Ag^+^ concentration of 1 μM, the AgNPs caused 36% cell death in *Escherichia coli*, which increased to 66% and 85% in the case of the AgNPs–H_3_PW_12_O_40_ and the Ag-NPs–H_3_PMo_12_O_40_ system, respectively, whereas both pristine POMs did not show antibacterial activity. Similar observations were made in the case of *Staphylococcus albus* but to a lesser extent indicating a lower antibacterial activity against Gram-positive bacteria (31%, 49% and 57% bacterial cell death, respectively). The antibiotic activity of Ag^+^ seems to potentiate the action of the investigated POMs as their synergy led to a remarkable increase in activity. It is suggested that these POMs are stabilized and transported into the bacterial cells by the AgNPs as Ag^+^ is able to disrupt the cell wall under the production of high concentration of reactive oxygen species (ROS). Furthermore, the nanoparticles did not exhibit toxicity against human cells (tested on the human prostate cancer cell line PC3).

The same two POMs, H_3_PW_12_O_40_ and H_3_PMo_12_O_40_, exhibited similar effects with gold nanoparticles (AuNPs) against *Escherichia coli*, although AuNPs are normally biocompatible with this bacterium.^83^ Surface functionalization of the AuNPs with POMs turned them into strong antibacterially active agents as bacterial cell death was increased from 7% to 43% in the case of H_3_PW_12_O_40_ and to 49% in the case of H_3_PMo_12_O_40_. The antibacterial activity was even further enhanced by modification of the AuNP–POM systems with cationic lysine leading to cell death rates of 75% (POT) and 96% (POMo), respectively. In contrast to the AgNP systems, POMo functionalized AuNPs showed higher bioactivity than the corresponding POT-functionalized particles due to a higher contentment of both gold and lysine in the former system. The significant improvement in activity by lysine modification can be attributed to its cationic nature, which most likely guides the nanomaterial towards the negatively charged cell walls and thus increases the interaction rate between the material and the bacterial cells. The same experiment against Gram-positive bacteria was not undertaken as the unmodified AuNPs were already too active on *Staphylococcus albus* leading to almost complete bacterial cell death.^83^

### 4.3. Amino acid–/peptide–POM nanocomposites

**Fig. 6:**
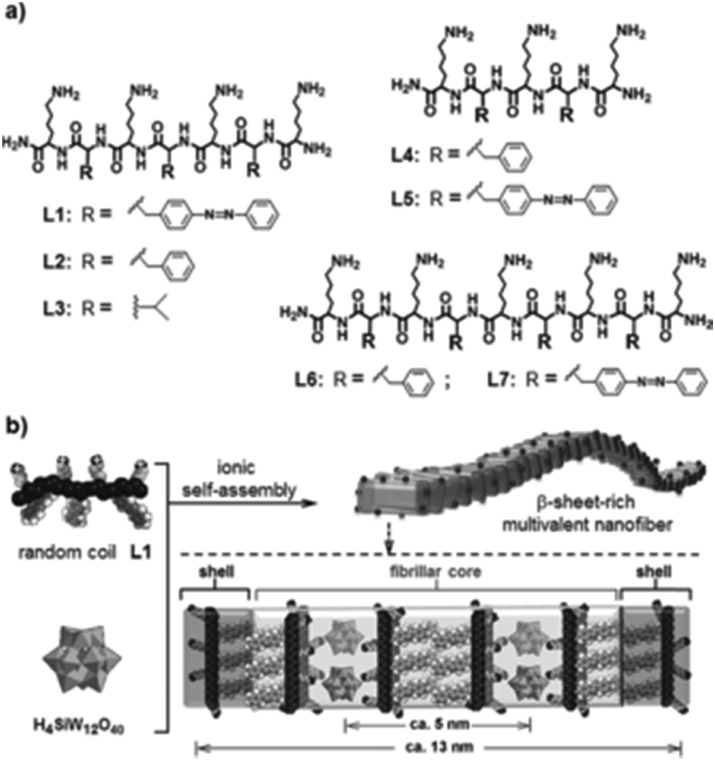
Structure of the peptide–POM nanocomposite.^86^ (a) Structure of the cationic peptides L1–L7, which were used in the study. (b) Ionic self-assembly of peptide L1 and H_4_SiW_12_O_40_. The figure is reprinted from Li *et al.* (2016)^86^ with permission from the copyright holder (Wiley).

Compounds having amino acid and/or peptide moieties are known to possess biological and pharmacological activities, among them also antibacterial activity.^84^ Therefore, the association between amino acids/peptides and POMs is a good strategy to improve their antibacterial properties. Three nanorod-amino acid phosphomolybdates, (HGly)_3_[PMo_12_O_40_](Gly)_9_ (Gly = glycine), (HLys)_9_[PMo_12_O_40_](Lys)_4_ (Lys = lysine) and (HHis)_3_[PMo_12_O_40_](His)_3_ (His = histidine), were reported to have remarkable antibacterial activity against *Escherichia coli* as they produced inhibition zones of ∼1.2–1.3 cm on solid growth agar plates.^85^ The Keggin POM and the amino acids themselves had only very weak antibacterial activity (inhibition zone of ∼0–0.7 cm) indicating a significant synergy effect, which could be mainly attributed to the nano-size effect rather than to *e.g.* electrostatic aspects as all three compounds were similarly active independent of the used amino acids. Li *et al.* demonstrated the POM-driven ([SiW_12_O_40_]^4–^) self-assembly of short peptides into multivalent nanofibers (Fig. 6).^86^ The nanofibers exhibited high surface areas and concentrated positive charges, which resulted in antibacterial activity against *Escherichia coli* (MIC = 60 μM). The constituents themselves had only poor activity. The bacterial growth inhibiting effect was suggested to be based on the electrostatic binding of the cationic peptides to the cell membrane, on which surface the peptides are accumulated before they enter the cell in order to induce cell lysis. However, it cannot be excluded that in this case the role of the POM was solely the initiation of the self-assembly of the nanofibers and the subsequent stabilization of the suprastructure with no direct antibacterial contribution.

### 4.4. Other POM-based nanocomposites

Nanohybrid membranes consisting of the Keggin POM H_5_PV_2_Mo_12_O_40_ and poly(vinylalcohol)/polyethylenimine (PVA/PEI) exhibited antibacterial activity against the Gram-positive bacteria *Staphylococcus aureus* and *Bacillus subtilis* and the Gram-negative *Escherichia coli* and *Pseudomonas aeruginosa*.^87^ The bioactivity increased with increasing H_5_PV_2_Mo_12_O_40_ content within the PVA/PEI membrane exhibiting MIC values in the range of 0.02–2 μg ml^–1^ for the Gram-positive and 0.2–2 μg ml^–1^ for the Gram-negative bacteria (Table S4, ESI†). Another promising nanocomposite system is that of the same Keggin structure H_5_PV_2_Mo_12_O_40_ and bamboo charcoal (BC).^88^ BC is a kind of carbon-based material and one of the most used adsorbents in extraction studies. The POM–BC system was strongly active against seven bacterial strains, including both Gram-positive and -negative strains, in a POMo-concentration dependent manner (Table S4, ESI†). The nanocomposite with the highest POMo content (weight ratio of BC : POMo = 1 : 3) exhibited a MIC of 4 μg ml^–1^ against all tested bacteria. Even antibiotic resistant strains like the ciprofloxacin-resistant strain of *Pseudomonas aeruginosa* (CRPA) were inhibited and at higher composite concentrations (16 μg ml^–1^ of the most highly POMo-loaded composite) even completely killed as evidenced by morphological changes of the respective bacteria. Very recently, a series of compounds composed of POM and phosphonium groups (MePh_3_P) showed also antibacterial activity against both *Staphylococcus aureus* and *Escherichia coli*, which was even stronger than that of the antibiotic ampicillin as judged by the size of the inhibitory zones on agar plates.^89^ The (MePh_3_P)_*n*_–POM systems (*n* = 3 or 4) are based on the Keggin-type POMs [SiW_12_O_40_]^4–^, [PW_12_O_40_]^3–^ or [PMo_12_O_40_]^3–^ and the quaternary phosphonium salt methyl triphenyl phosphonium iodide (MePhPI). It was described that the antibacterial activity increased with increasing content of crystal water within the structures. This was attributed to the hydrophilic affinity between the compounds and the bacterial cell wall as the latter contains hydrophilic constituents. However, the main factor for the antibacterial activity of these compounds was the increase of the positive charge of the phosphonium cations due to the POM-induced polarization, which increases the electrostatic force between the compounds and the bacterial cell membrane.

Multilayer films based on the Keggin POMs [SiW_12_O_40_]^4–^ and [PMo_12_O_40_]^3–^ and the dye methylene blue were reported to be active against *Escherichia coli*.^90^ Methylene blue as being involved in the electron transport of different biological processes is a natural antibiotic and is used in the photodynamic therapy of bacteria, fungi, viruses and cancer.^91^ However, a control experiment with a PEI-film containing only methylene blue exhibited negligible activity and therefore the antibacterial activity of the methylene blue/POMo film could be, at least partially, ascribed to the Keggin-POMo suggesting some kind of synergy. A similar study with a layer-by-layer film of [H_4_PV_6_Mo_6_O_40_]^5–^ and methyl violet, which is a strong bactericide, showed the same result as in the case of methyl blue as only the POM–dye hybrid film performed antibacterial activity against *Escherichia coli*, whereas the control film lacking POM did not.^92^

Very recently, the antibacterial potential of POM-ionic liquids (POM-ILs) were reported.^93^,^94^ The studied POM-ILs were composed of the lacunary Keggin-POT [SiW_11_O_39_]^8–^ (Fig. 1F) and three antimicrobial tetraalkylammonium cations differing in their alkyl chain length. All three POM-ILs were active against the tested bacteria, namely *Staphylococcus aureus*, *Pseudomonas aeruginosa* and *Escherichia coli*, whereby *Staphylococcus aureus* was particularly sensitive to the POM-ILs. The antibacterial activity of these nanocomposites somehow correlated with the alkyl chain length of the cations as POM-ILs bearing cations with C7 and C8 alkyl chains (MIC values of 2–100 μg ml^–1^) were significantly more active than their counterparts possessing C6 chained cations (MIC values of 10–1000 μg ml^–1^) (Table S4, ESI†).

The effects of the above described POM-hybrid and nanostructure on all tested bacteria are summarized in Table 2.

**Table 2:** Overview of the effect of POM-based hybrids and nanocomposites on all tested bacteria

POM-hybrid/nanocomposite	Antibacterial active on	Ref.	POM-hybrid/nanocomposite	Antibacterial active on	Ref.
Organoantimony-polyoxotungstates:	*B. subtilis* ^(+)^	61–64	PVA/PEI–POM:	*S. aureus* ^(+)^	87
	*C. michiganensis* ^(+)^	62–64		*B. subtilis* ^(+)^	87
	*Paenibacillus* sp.^(+)^	62–64		*E. coli* ^(–)^	87
	*E. coli* ^(–)^	61–64		*P. aeruginosa* ^(–)^	87
	*P. putida* ^(–)^	62–64			
	*Vibrio* sp.^(–)^	62–64	Peptide–POM:	*E. coli* ^(–)^	85 and 86

Gatifloxacin–POM:	*S. aureus* ^(+)^	65	Bamboo charcoal–POM:	*S. aureus* ^(+)^	88
	*E. coli* ^(–)^	65		MR *S. aureus*^(+)^	88
				*B. subtilis* ^(+)^	88
Zn–pipemidic acid–Zn–POM:	*S. aureus* ^(+)^	66		*E. coli* ^(–)^	88
	*E. coli* ^(–)^	66		*P. aeruginosa* ^(–)^	88
				CR *P. aeruginosa*^(–)^	88
(C_9_H_8_N)_3_[NbW_5_O_19_]:	*S. agalactiae* ^(+)^	67			
	*L. acidophilus* ^(+)^	67	MePh_3_P–POMs:	*S. aureus* ^(+)^	89
	*E. coli* ^(–)^	67		*E. coli* ^(–)^	89
	*S. typhimurium* ^(–)^	67			

Chitosan–POMs:	*S. aureus* ^(+)^	49	Multilayer films:	*E. coli* ^(–)^	90 and 92
	*E. coli* ^(–)^	49 and 75–77			

Ag/AuNPs–POM:	*S. albus* ^(+)^	82 and 83	POM ionic liquids:	*S. aureus* ^(+)^	94
	*E. coli* ^(–)^	82 and 83		*E. coli* ^(–)^	94
				*P. aeruginosa* ^(–)^	94

## 5. Structure–activity-relationship of antibacterially active POMs and their putative mechanisms

### 5.1. Structure–activity-relationship

**Fig. 7:**
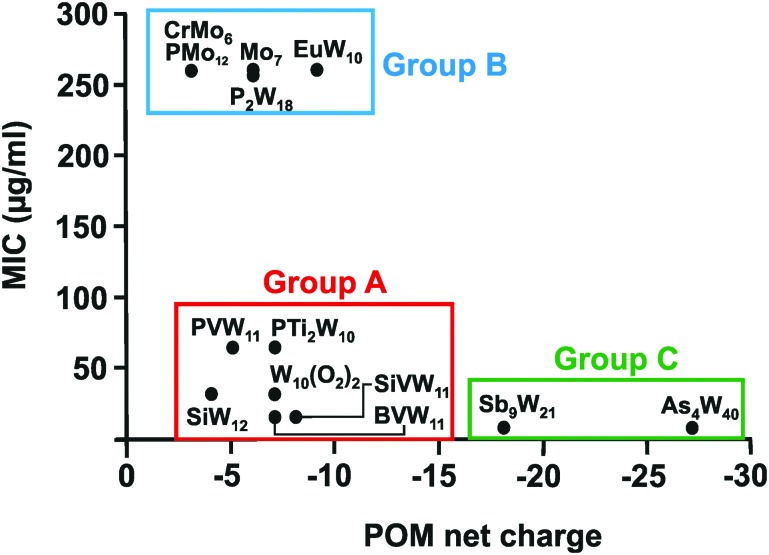
Charge–activity-relationship of POMs against the metronidazole-resistant strain IID3023 of *Helicobacter pylori*. The net charges of the POMs are plotted against their MIC values. Specific groups are marked, namely group A (red) of POMs with higher activity (MIC up to 100 μg ml^–1^), group B (blue) of POMs with lower activity (MIC > 200 μg ml^–1^) and group C (green) of large and highly charged POMs exhibiting the highest activity. CrMo_6_ = [CrMo_6_O_24_H_6_]^3–^, PMo_12_ = [PMo_12_O_40_]^3–^, Mo_7_ = [Mo_7_O_24_]^6–^, EuW_10_ = [EuW_10_O_36_]^9–^, P_2_W_18_ = [P_2_W_18_O_62_]^6–^, PVW_11_ = [PVW_11_O_40_]^5–^, PTi_2_W_10_ = [PTi_2_W_10_O_40_]^7–^, W_10_(O_2_)_2_ = [PTi_2_W_10_O_38_(O_2_)_2_]^7–^, SiW_12_ = [SiW_12_O_40_]^4–^, SiVW_11_ = [SiVW_11_O_40_]^7–^, BVW_11_ = [BVW_11_O_40_]^7–^, Sb_9_W_21_ = [KSb_9_W_21_O_86_]^18–^, As_4_W_40_ = [KAs_4_W_40_O_140_]^27–^.

In total 74 inorganic POMs were tested on the MRSA strains SR3605 and ATCC43300 (Table S1, ESI†), where only seven compounds showed considerable antibacterial activity by themselves (MIC up to 100 μg ml^–1^). The most effective ones were double Keggin, Keggin-type and Wells–Dawson-type structures. However, most of the tested POTs exhibited significant antibacterial activity in synergy with β-lactam antibiotics. Analysis of the structure–activity-relationship considering the parameters POM size (number of addenda atoms), POM total net charge, POM charge density (charge per number of addenda atoms), number of certain atoms within the POM (*e.g.* different metals and oxygen atoms) and POM redox potential did neither reveal any unambiguous correlation nor any trend or structure-function pattern for these POMs. The same was also observed for the synergy effect, where no correlation between the POM-mediated sensitizing effect and its structure was found. However, considering the biological activity of the so far studied POMs against *Helicobacter pylori*, structure–activity patterns were identified (Table 3 and Fig. 7). The charge–activity-relationship of all tested POMs against the IID3023 strain of *Helicobacter pylori* was determined (Fig. 7).

**Table 3:** Antibacterial activity of POMs alone (MIC) against drug-susceptible and metronidazole- and clarithromycin-resistant *H. pylori* strains

POM	MIC (μg ml^–1^)								
	DSS^*a*^	Hp	Hp	Hp	IID	ATCC	Hp	Hp	Ref.
		018^*b*^	030^*b*^	065^*b*^	3023^*b*^	43504^*b*^	027^*c*^	067^*c*^	
Polyoxotungstate:									
Keggin:									
H_4_[SiW_12_O_40_]	16–256	128	32	64	32	64	256	64	46
K_7_[PTi_2_W_10_O_40_]	64–256	256	128	128	64	128	256	64	46
(NH_3_Pr^*i*^)_6_H[PTi_2_W_10_O_38_(O_2_)_2_]	32–256	64	64	32	32	64	256	64	46

Wells–Dawson:									
K_6_[P_2_W_18_O_62_]	256 to >256	128	n.d.^*d*^	256	256	256	256	256	46

Decatungstate:									
Na_9_[EuW_10_O_36_]	>256	>256	n.d.^*d*^	>256	>256	>256	>256	>256	46

Other structure:									
K_27_[KAs_4_W_40_O_140_]	16	16	16	16	8	8	8	32	46
K_18_[KSb_9_W_21_O_86_]	16–64	16	64	16	8	16	8	32	46

Polyoxovanadotungstate:									
Keggin:									
K_5_[PV^IV^W_11_O_40_]	16–256	32	64	128	64	32	256	128	46
K_5_[SiV^V^W_11_O_40_]	16–256	64	16	16	16	64	64	64	46
K_7_[BV^IV^W_11_O_40_]	32–256	32	16	128	16	32	128	64	46

Polyoxomolybdate:									
Keggin:									
H_3_[PMo_12_O_40_]	>256	>256	n.d.^*d*^	>256	>256	>256	>256	>256	46

Anderson-Evans:									
Na_3_[CrMo_6_O_24_H_6_]	>256	>256	>256	>256	>256	>256	>256	>256	46

Heptamolybdate:									
(NH_4_)_6_[Mo_7_O_24_]	>256	>256	>256	>256	>256	>256	>256	>256	46

Antibiotic:									
Amoxicillin	0.001–0.063	0.008	0.032	0.063	0.016	0.032	2	0.25	46
Clarithromycin	0.001–0.125	0.032	0.004	16	0.032	0.032	32	256	46
Metronidazole	0.5–4	32	16	128	128	128	4	2	46

^*a*^Ten drug-susceptible strains (DSS) of *H. pylori* were tested and the MIC range is given. No further information about the exact strains is given.

^*b*^Hp018, Hp030, Hp065, IID3023 and ATCC43504 are metronidazole (MTZ)-resistant *H. pylori* strains.

^*c*^Hp027 and Hp067 are clarithromycin (CLR)-resistant *H. pylori* strains.

^*d*^No data available. NH_3_Pr^i^ = isopropylammonium.

From Fig. 7 it can be deduced that POMs possessing a net charge between –2 to –8 can be divided into two groups, namely POMs exhibiting MIC values up to 100 μg ml^–1^ (group A) and those possessing MICs over 200 μg ml^–1^ (group B). Among POMs belonging to group A, vanadium-containing POMs showed the highest activity. POMs exhibiting a negative charge higher than –15 showed the highest activity against almost all *Helicobacter pylori* strains (one exception: strain Hp030, Fig. S4, ESI†) as indicated by MIC values < 50 μg ml^–1^ (group C).

**Fig. 8:**
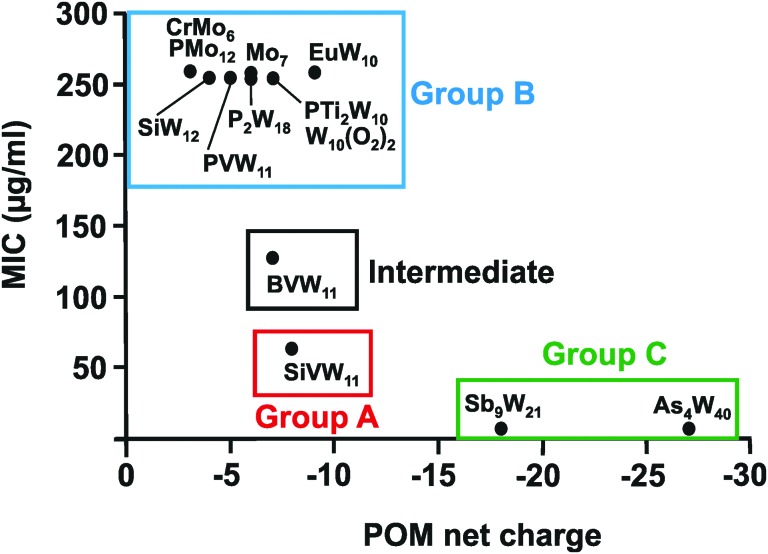
Charge–activity-relationship of POMs against the clarithromycin-resistant strain Hp027 of *Helicobacter pylori*. The net charges of the POMs are plotted against their MIC values. Specific groups are marked, namely group A (red) of POMs with higher activity (MIC up to 100 μg ml^–1^), group B (blue) of POMs with lower activity (MIC > 200 μg ml^–1^) and group C (green) of large and highly charged POMs exhibiting the highest activity. Between MIC values of 100 and 200 μg ml^–1^ there is an intermediate zone (black) representing moderately active POMs. For full POM formula, see caption of Fig. 7.

The same or similar trends were observed for 16 of the 17 tested *Helicobacter pylori* strains (Fig. S2 to S14, ESI†). The only strain not following this pattern was the clarithromycin-resistant Hp027, where most of the POMs belonging to group A (MIC values up to 100 μg ml^–1^ in most of the strains) shifted towards lower activity and thus to group B (MIC >200 μg ml^–1^) (Fig. 8). Only the vanadium-containing [SiVW_11_O_40_]^8–^ and the two large and highly charged POMs from group C maintained their high activity in this strain. The Wells–Dawson anion [P_2_W_18_O_62_]^6–^ was the most strain sensitive POM as depending on the strain its antibacterial activity was fluctuating the most (from lower to intermediate activity).

**Fig. 9:**
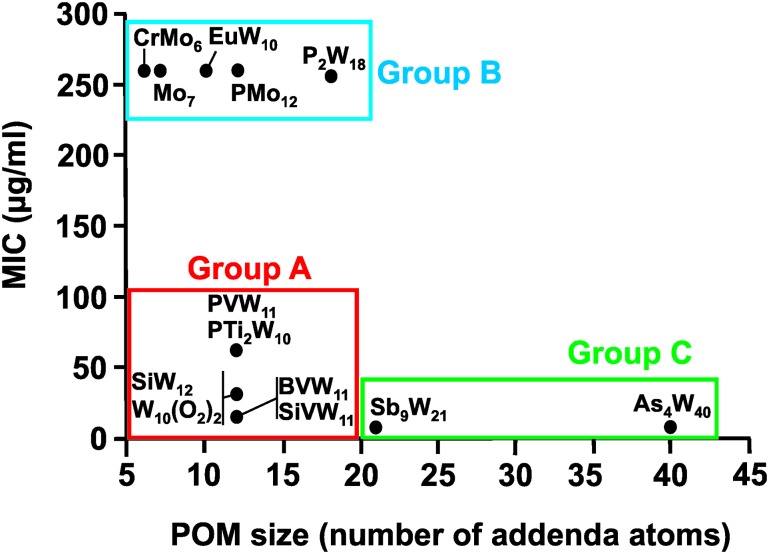
Size–activity-relationship of POMs against the metronidazole-resistant strain IID3023 of *Helciobacter pylori*. Specific groups are marked, namely group A (red) of POMs with higher activity (MIC up to 100 μg ml^–1^), group B (blue) of POMs with lower activity (MIC > 200 μg ml^–1^) and group C (green) of large and highly charged POMs exhibiting the highest activity. For full POM formula, see caption of Fig. 7.

Regarding the POM size *vs.* activity correlation, where the size is defined as the total number of addenda atoms, a similar pattern as described above for the charge was observed (Fig. 9). Thus, POMs exhibiting a size from 6 to 22 addenda atoms can also be subdivided into group A (MIC up to 100 μg ml^–1^) and group B (MIC > 200 μg ml^–1^), whereas the huge and highly charged [KSb_9_W_21_O_86_]^18–^ and [KAs_4_W_40_O_140_]^27–^ form again their own group C (MIC < 50 μg ml^–1^). Within both the charge–activity- and size–activity-relationship, there are some POMs showing intermediate activities (MIC value between 100 and 200 μg ml^–1^), for example, [BVW_11_O_40_]^7–^ against the strain Hp027 (Fig. 8). According to the above observations, *Helicobacter pylori* is most sensitive to large and highly negatively charged POMs but also susceptible to Keggin-type POMs with vanadium-substituted structures being among the most effective ones. On the other hand, the least active POMs were in general POMos and POMs with other than Keggin-type structures like the Anderson-Evans and decatungstate structure (Fig. 8 and Fig. S2–S14, ESI†).

**Fig. 10:**
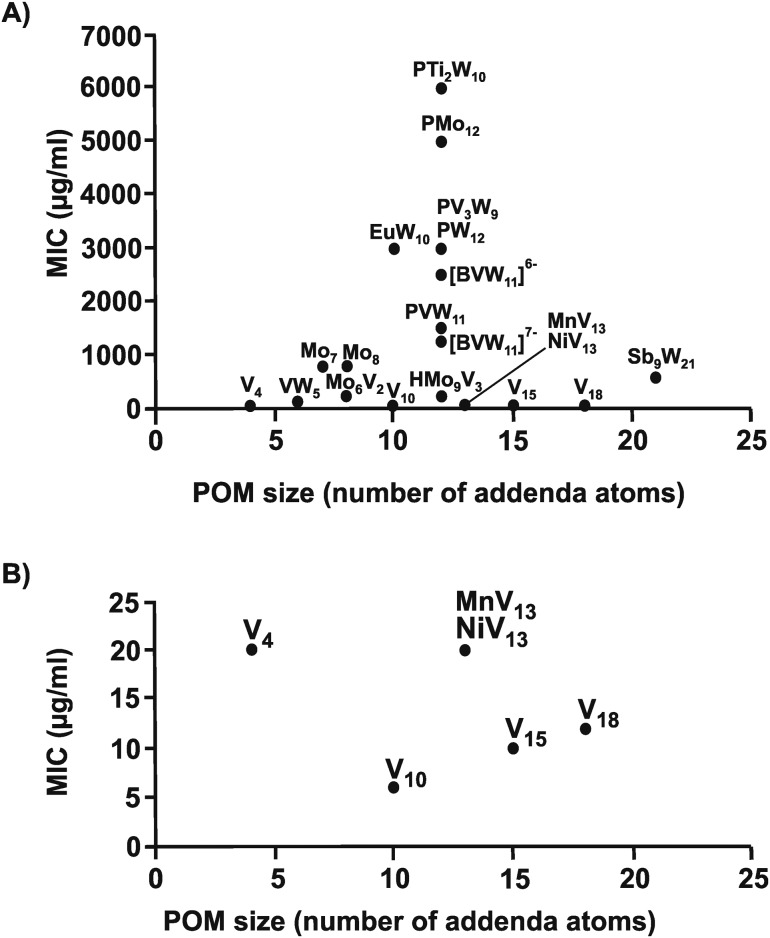
Size–activity-relationship of POMs against six strains of *Streptococcus pneumoniae*. (A) The sizes of the POMs expressed as number of addenda atoms are plotted against their MIC values. (B) Zoom view of (A) by plotting the MIC value only in the range of 0 to 25 μg ml^–1^. PMo_12_ = [PMo_12_O_40_]^3–^, Mo_6_V_2_ = [Mo_6_V_2_O_26_]^6–^, HMo_9_V_3_ = [HMo_9_V_3_O_38_]^6–^, Mo_7_ = [Mo_7_O_24_]^6–^, Mo_8_ = [Mo_8_O_26_]^4–^, EuW_10_ = [EuW_10_O_36_]^9–^, PVW_11_ = [PVW_11_O_40_]^5–^, PTi_2_W_10_ = [PTi_2_W_10_O_40_]^7–^, PW_12_ = [PW_12_O_40_]^3–^, [BVW_11_]^6–^ = [BV^V^W_11_O_40_]^6–^, [BVW_11_]^7–^ = [BV^IV^W_11_O_40_]^7–^, PV_3_W_9_ = [PV_3_W_9_O_40_]^6–^, Sb_9_W_21_ = [KSb_9_W_21_O_86_]^18–^, V_4_ = [V_4_O_12_]^4–^, VW_5_ = [VW_5_O_19_]^4–^, V_10_ = [V_10_O_28_]^6–^, MnV_13_ = [MnV_13_O_38_]^7–^, NiV_13_ = [NiV_13_O_38_]^7–^, V_15_ = [V_15_O_36_(CO_3_)]^7–^, V_18_ = [V_18_O_42_(H_2_O)]^12–^.

**Fig. 11:**
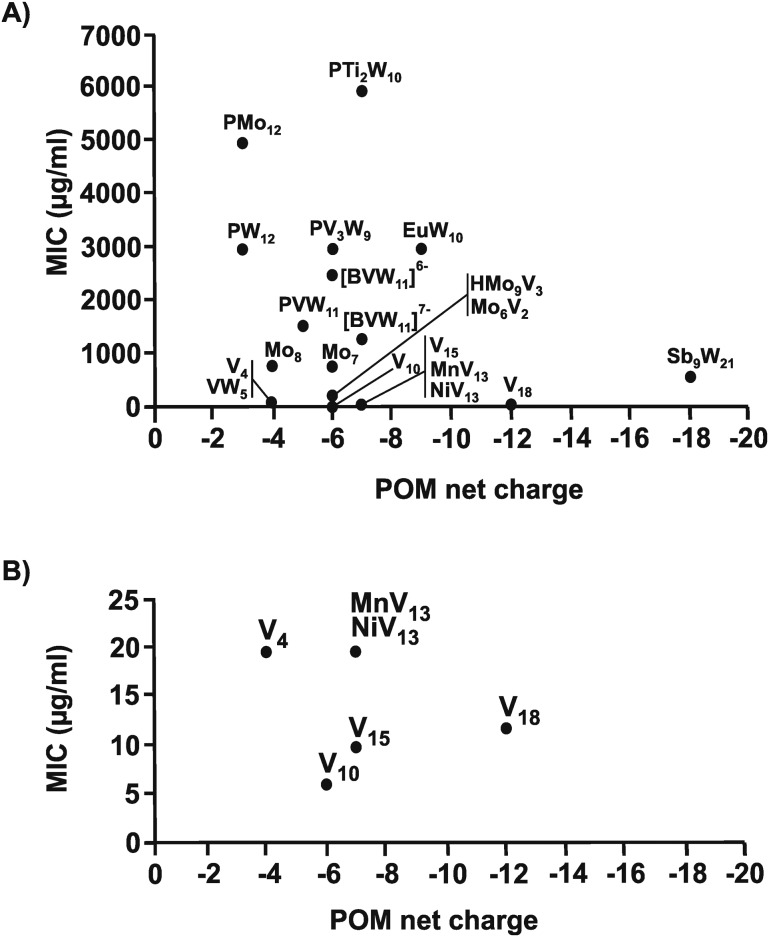
Charge–activity-relationship of POMs against six strains of *Streptococcus pneumoniae*. (A) The sizes of the POMs expressed as number of addenda atoms are plotted against their MIC values. (B) Zoom view of (A) by plotting the MIC value only in the range of 0 to 25 μg ml^–1^. For POM sum formula, see caption of Fig. 10.

The same structure–activity analysis was performed for *Streptococcus pneumoniae* revealing that this bacterium is especially sensitive to POVs with decavanadate exhibiting the highest antibacterial activity (Fig. 10 and 11). In contrast to *Helicobacter pylori*, most of the tested POMos were determined to be more active than POTs (Table S3, ESI†). Furthermore, as can be seen in Fig. 10 and 11, isostructural POMs (Keggin-type) present completely different antibacterial activity with MIC values ranging from 192 μg ml^–1^ for [HMo_9_V_3_O_38_]^6–^ to 6000 μg ml^–1^ for [PTi_2_W_10_O_40_]^7–^ indicating the generally low response of this bacterium towards this POM-type. According to our analysis, the activity of POMs are highly bacteria-dependent as different bacteria react distinctly to certain types of metal species and it seems that there is a certain threshold for the parameters charge, size and shape (POM-type) determining the biological activity of POMs. However, decavanadate seems to be special in this regard as it exhibits promising antibacterial activity against the majority of the tested bacteria, which most likely is connected to its putative mechanisms of action as its biological behavior and activity is well studied not only *in vitro* but also *in vivo*.^38^,^40^,^95^–^101^ However, the susceptibility of certain bacterial strains (*e.g. Streptococcus pneumoniae*) towards vanadium-containing POMs could be also indicative of the involvement of redox-processes in the killing process as vanadium is most readily reduced, among the typical addenda atoms (W, Mo and V), leading to an increase in the redox activity of the corresponding POMs.^102^ Another function-activity-relationship was already mentioned in Section 3.1, where a series of organoantimony(iii)-containing POTs showed a direct correlation between their antibacterial activity and the amount of attached organic moieties.

Note, that it is in general difficult to deduce structure–activity correlations for POMs as their stability is a critical issue and therefore the identity of the active species is unknown for most POMs, especially in cases, where the POMs have been analyzed in bacterial growth media. In addition to this, the available data describing the antibacterial activity of POMs is more or less biased as the vast majority of the tested compounds are Keggin-based structures leading to a clustering of data in favor of this archetype.

### 5.2. Putative mode of actions

**Fig. 12:**
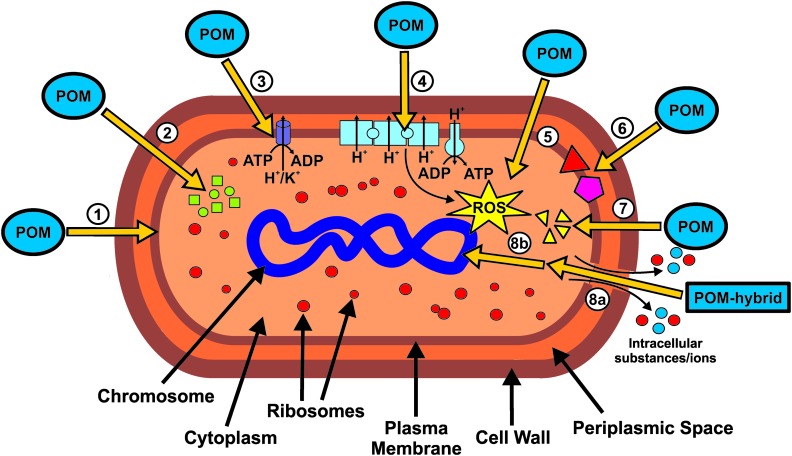
Schematic overview of the putative mechanisms of antibacterially active POMs. POMs are uptaken into the inner membrane but not into the cytoplasm. POMs inhibit the production of both PBP2a (green squares) and β-lactamases (green circles). POMs, especially decavanadate, target P-type ATPases. Impairment of the bacterial electron-transport-chain (respiratory system) by POMs. POM-mediated increase of the ROS-level *via* oxidation. POMs can also react with important membrane anchored proteins and enzymes (red triangle and purple pentagon). The disruption of the bacterial cytoskeleton dynamics by POM-interactions with cytoskeletal elements (yellow triangles). (8a) POM-based nanocomposites (*e.g.* CT–POMs or AgNP–POMs) are able to disrupt the bacterial cell wall leading to leakage of intracellular substances (blue and red circles). (8b) Upon cell wall disruption, POMs could interact with cytoplasmic elements or proteins that are anion-sensitive like nucleic acid binding proteins.

The putative mechanisms of action of POMs with antibacterial activity can be briefly resumed in the scheme of Fig. 12. The POM targets include extracellular- and membrane-associated proteins/enzymes and processes such as respiration^96^ (or other redox processes) or cytoskeleton dynamics.^39^,^56^ POMs were successfully localized within or at the inner membrane of bacteria, suggesting the interference or interaction with the bacterial cell wall and membrane compounds (Fig. 12, ).^46^,^48^ There is no report about antibacterial POMs reaching the cytoplasm, however, some suggested mechanisms would highly benefit from POMs being active within the cytoplasmic space. There are some theories suggesting that POMs (without considering hybrid or nanocomposite structures) could be internalized by some surface proteins *via* endocytosis-like processes, for example, macrophage receptors as it was proposed that POTs do compete with their ligand, acetylated low density lipoprotein, for receptor binding.^103^ Depending on the POM type, the transcription or translation or even both processes of PBP2a are reduced.^27^ In addition, POMs inhibit the production of β-lactamases.^43^ Thus, POMs are able to interfere with two proteins that are responsible for the β-lactam resistance of some bacteria (Fig. 12, ). However, the exact mode of action is unknown but POMs might interact with protein targets, which are part of a signal cascade necessary for the production of the aforementioned proteins. POMs, especially decavanadate, were shown to be potent inhibitors of P-type ATPases and thus it is suggested that, at least partially, the malfunction of ATPases is also responsible for the observed antibacterial effects.^40^ Inhibition of P-type ATPases has a severe impact on the cellular metabolism (Fig. 12, ). As most POMs are highly redox-active, they could impair the bacterial electron-transport-chain (respiratory system) by oxidizing important electron carriers like NADH and thus affect ATP production.^98^,^104^ This in turn leads to lethal damage in bacterial cells (Fig. 12, ). One aftermath of impairing the respiratory system is the increase of the ROS level. However, POMs can also directly produce ROS by oxidizing proteins, lipids and other bacterial compounds (Fig. 12, ). In addition, POMs are able to elevate the ROS level by oxidizing the antioxidant glutathione (GSH), which leads to the depletion of the GSH-pool.^105^ POMs can also react with important membrane anchored proteins and enzymes, which could lead to serious damage within the bacterial cell. For example, some POMs are able to inhibit sialyl- and sulfotransferases and thus could interfere with the bacterial carbohydrate metabolism (Fig. 12, ).^106^ Another possible mechanism is the disruption of the bacterial cytoskeleton dynamics by POM-interactions with cytoskeletal elements like FtsZ and MreB (Fig. 12, ). Some POM-based nanocomposites like CT–POMs or AgNP–POMs are able to disrupt the bacterial cell wall, which leads to the leakage of intracellular substances (Fig. 12, 8a).^74^,^79^ Once the cell wall is disrupted, POMs could interact with cytoplasmic elements or proteins that are anion-sensitive like nucleic acid binding proteins. On the other hand, other constituents of the hybrid system like CT can directly interact with DNA molecules and thus causing lethal damages to the bacteria (Fig. 12, 8b).^74^

In general, it seems that POM–protein interactions are mainly responsible for the antibacterial activity of the inorganic compounds. As these interactions are of electrostatic nature, almost every protein is a potential target for POMs as they normally bear positively charged regions. Besides the (possibly) reversible electrostatic interactions, POMs are also able to covalently bind to proteins.^6^,^35^ This demonstrates the wide applicability of POMs in biological fields. Due to the POM's ability to inhibit a series of biologically relevant proteins and enzymes, the mode of action of antibacterially active POMs might not be explained by one strict mechanism with one single target but rather by multiple POM–protein interactions affecting several biological pathways at the same time and the sum of these disturbances ultimately leads to the death of the bacterial cell.^6^

In the next years, we expect that important questions will be answered: (1) What is the exact mechanism of POM uptake? (2) Where is the exact location of the POM and (3) its action within the cell? (4) What is the exact bioactive POM species and how does it function (mode of action)? (5) What is the exact mechanism of POM-induced bacterial resistance reversion? These and other questions will require continuous development of new techniques and approaches to explore the effects of POMs on bacteria and their medicinal applications in more detail.

## 6. Conclusions and future perspective

The high potential of POMs, especially POM-based hybrid systems, as antibacterial agents has been proved by the research activities of the last two decades. POMs have been shown to be active on both Gram-negative and Gram-positive bacteria, among them also highly hazardous antibiotic resistant bacterial strains, and were partially more active than commonly used antibiotics. POMs were shown to exhibit great synergy with some conventional antibiotic agents, which is especially important for the treatment of highly resistant bacteria, but also direct antibacterial activity was observed. An analysis of the structure–activity-relationship of a series of POMs against two bacteria, namely *Helicobacter pylori* and *Streptococcus pneumoniae*, indicated that isostructural POMs, despite having the same size and similar charge, can be split into two groups according to their activity. Regarding *Helicobacter pylori*, POMs exhibiting the highest activity were mostly Keggin-type POTs, polyoxovanadotungstates and large highly negatively charged POMs, whereas in the case of *Streptococcus pneumoniae*, the most active POMs were ascribed to be POVs, especially decavanadate, which was also very active against other bacteria. Despite the considerable potential of POMs as metallodrugs, applications of inorganic POMs in medicinal fields are prevented by both their toxicity and the lack of knowledge about their mode of action. The former issue can be avoided by formation of hybrid structures, which were shown to drastically reduce the toxicity arising from the pristine POM. Nevertheless, more studies are needed to explore the biological effects of POMs that causes their toxicity. Furthermore, comprehensive investigations on more bacteria using more quantitative methods have to be performed to better decipher the structure–activity-relationship of bioactive POMs. For this reason, it would be beneficial to establish a standard method to normalize the evaluation of POM-mediated antibacterial activity. It also requires the step up of the research effort to tackle the problem regarding the mode of antibacterial action of POMs as both bacterial growth inhibition and cell death are associated with a great cascade of reactions making it enormously challenging to pinpoint the exact inhibitory event. Therefore, interdisciplinary collaborations are needed to collect and accumulate contributions from different research fields like inorganic chemistry (POM chemistry), pharmacy/medicinal chemistry (antibacterial effect of POMs), crystallography (structure of POM-target complexes) and biochemistry (location of POM action). Future research will be focused on the development of novel hybrid compounds with enhanced stability and biological activity and reduced toxicity. Besides investigating more bacteria strains, the detection of potential target enzymes and the analysis of their interaction with POMs will be at the top of the agenda of future research. As POMs were only found to be located at the periphery of bacterial cells, the search for potential targets should be shifted towards extracellular or membrane-associated proteins, which are accessible without the need of POM penetration into the cytoplasm. Furthermore, from a medical point of view, membrane proteins represent the most important group of proteins/enzymes as their functions are vital for the survival of organisms. In this way, structure–activity relationships can be elaborated enabling the targeted synthesis of powerful POM-based antibiotic compounds.

## Conflicts of interest

There are no conflicts to declare.

## Supplementary Material

Supplementary information

## References

[cit1] Pope M. T., Heteropoly and isopoly oxometalates, Springer-Verlag, Berlin, New York, 1983.

[cit2] Wang S.-S., Yang G.-Y. (2015). Chem. Rev..

[cit3] Papaconstantinou E. and Hiskia A., Polyoxometalate Molecular Science, Springer, Dordrecht, 2003, pp. 381–416.

[cit4] Viswanathan B., Environmentally Benign Catalysts, Springer, Dordrecht, 2013, pp. 245–255.

[cit5] Casañ-Pastor N., Gómez-Romero P. (2004). Front. Biosci..

[cit6] Bijelic A., Rompel A. (2015). Coord. Chem. Rev..

[cit7] Bijelic A., Rompel A. (2017). Acc. Chem. Res..

[cit8] Rhule J. T., Hill C. L., Judd D. A., Schinazi R. F. (1998). Chem. Rev..

[cit9] Arefian M., Mirzaei M., Eshtiagh-Hosseini H., Frontera A. (2017). Dalton Trans..

[cit10] Proust A., Matt B., Villanneau R., Guillemot G., Gouzerh P., Izzet G. (2012). Chem. Soc. Rev..

[cit11] Blazevic A., Al-Sayed E., Roller A., Giester G., Rompel A. (2015). Chem. – Eur. J..

[cit12] Al-Sayed E., Blazevic A., Roller A., Rompel A. (2015). Chem. – Eur. J..

[cit13] Blazevic A., Rompel A. (2016). Coord. Chem. Rev..

[cit14] Gumerova N. I., Roller A., Rompel A. (2016). Eur. J. Inorg. Chem..

[cit15] Gumerova N. I., Roller A., Rompel A. (2016). Chem. Commun..

[cit16] Izzet G., Volatron F., Proust A. (2017). Chem. Rec..

[cit17] Gumerova N. I., Krivosudský L., Fraqueza G., Breibeck J., Al-Sayed E., Tanuhadi E., Bijelic A., Fuentes J., Aureliano M., Rompel A. (2018). Metallomics.

[cit18] Chermann J. C., Raynaud M., Jasmin C., Mathé G. (1970). Nature.

[cit19] Inouye Y., Tokutake Y., Kunihara J., Yoshida T., Yamase T., Nakata A., Nakamura S. (1992). Chem. Pharm. Bull..

[cit20] Witvrouw M., Weigold H., Pannecouque C., Schols D., De Clercq E., Holan G. (2000). J. Med. Chem..

[cit21] Judd D. A., Nettles J. H., Nevins N., Snyder J. P., Liotta D. C., Tang J., Ermolieff J., Schinazi R. F., Hill C. L. (2001). J. Am. Chem. Soc..

[cit22] Nomiya K., Torii H., Hasegawa T., Nemoto Y., Nomura K., Hashino K., Uchida M., Kato Y., Shimizu K., Oda M. (2001). J. Inorg. Biochem..

[cit23] Hasenknopf B. (2005). Front. Biosci..

[cit24] Ilyas Z., Saeed Shah H., Al-Oweini R., Kortz U., Iqbal J. (2014). Metallomics.

[cit25] Treviño S., Velázquez-Vázquez D., Sánchez-Lara E., Diaz-Fonseca A., Flores-Hernandez J. Á., Pérez-Benítez A., Brambila-Colombres E., González-Vergara E. (2016). Oxid. Med. Cell. Longevity.

[cit26] Yamase T., Fujita H., Fukushima K. (1988). Inorg. Chim. Acta.

[cit27] Yamase T. (2005). J. Mater. Chem..

[cit28] Tajima Y., Nagasawa Z., Tadano J. (1993). Microbiol. Immunol..

[cit29] Turner T. L., Nguyen V. H., McLauchlan C. C., Dymon Z., Dorsey B. M., Hooker J. D., Jones M. A. (2012). J. Inorg. Biochem..

[cit30] Mauracher S. G., Molitor C., Al-Oweini R., Kortz U., Rompel A. (2014). Acta Crystallogr., Sect. F: Struct. Biol. Commun..

[cit31] Mauracher S. G., Molitor C., Al-Oweini R., Kortz U., Rompel A. (2014). Acta Crystallogr., Sect. D: Biol. Crystallogr..

[cit32] Bijelic A., Molitor C., Mauracher S. G., Al-Oweini R., Kortz U., Rompel A. (2015). ChemBioChem.

[cit33] Molitor C., Mauracher S. G., Rompel A. (2015). Acta Crystallogr., Sect. F: Struct. Biol. Commun..

[cit34] Molitor C., Mauracher S. G., Rompel A. (2016). Proc. Natl. Acad. Sci. U. S. A..

[cit35] Molitor C., Bijelic A., Rompel A. (2016). Chem. Commun..

[cit36] Molitor C., Bijelic A., Rompel A. (2017). IUCrJ.

[cit37] Stephan H., Kubeil M., Emmerling F., Müller C. E. (2013). Eur. J. Inorg. Chem..

[cit38] Borges G., Mendonça P., Joaquim N., Coucelo J., Aureliano M. (2003). Arch. Environ. Contam. Toxicol..

[cit39] Ramos S., Moura J. J. G., Aureliano M. (2010). J. Inorg. Biochem..

[cit40] Aureliano M., Fraqueza G., Ohlin C. A. (2013). Dalton Trans..

[cit41] Tajima Y. (1997). J. Inorg. Biochem..

[cit42] Yamase T., Fukuda N., Tajima Y. (1996). Biol. Pharm. Bull..

[cit43] Fukuda N., Yamase T., Tajima Y. (1999). Biol. Pharm. Bull..

[cit44] Tajima Y. (2001). Biol. Pharm. Bull..

[cit45] Tajima Y. (2002). Biomed. Res..

[cit46] Inoue M., Segawa K., Matsunaga S., Matsumoto N., Oda M., Yamase T. (2005). J. Inorg. Biochem..

[cit47] Georgopapadakou N. H. (1993). Antimicrob. Agents Chemother..

[cit48] Inoue M., Suzuki T., Fujita Y., Oda M., Matsumoto N., Yamase T. (2006). J. Inorg. Biochem..

[cit49] Chen S., Wu G., Long D., Liu Y. (2006). Carbohydr. Polym..

[cit50] Fukuda N., Yamase T. (1997). Biol. Pharm. Bull..

[cit51] Aureliano M., Madeira V. M. (1994). Biochem. Biophys. Res. Commun..

[cit52] Yatime L., Buch-Pedersen M. J., Musgaard M., Morth J. P., Winther A.-M. L., Pedersen B. P., Olesen C., Andersen J. P., Vilsen B., Schiøtt B., Palmgren M. G., Møller J. V., Nissen P., Fedosova N. (2009). Biochim. Biophys. Acta, Bioenerg..

[cit53] Rouzaire-Dubois B., Dubois J. M. (1990). Toxicon.

[cit54] Pelin M., Boscolo S., Poli M., Sosa S., Tubaro A., Florio C. (2013). Mar. Drugs.

[cit55] Cabeen M. T., Jacobs-Wagner C. (2005). Nat. Rev. Microbiol..

[cit56] Marques M. P. M., Gianolio D., Ramos S., Batista de Carvalho L. A. E., Aureliano M. (2017). Inorg. Chem..

[cit57] Erickson H. P. (2001). Nature.

[cit58] Shaevitz J. W., Gitai Z. (2010). Cold Spring Harbor Perspect. Biol..

[cit59] Bae E., Lee J. W., Hwang B. H., Yeo J., Yoon J., Cha H. J., Choi W. (2008). Chemosphere.

[cit60] Tajima Y., Nagasawa Z., Tanabe I., Kusaba K., Tadano J. (1994). Microbiol. Immunol..

[cit61] Barsukova-Stuckart M., Piedra-Garza L. F., Gautam B., Alfaro-Espinoza G., Izarova N. V., Banerjee A., Bassil B. S., Ullrich M. S., Breunig H. J., Silvestru C., Kortz U. (2012). Inorg. Chem..

[cit62] Yang P., Bassil B. S., Lin Z., Haider A., Alfaro-Espinoza G., Ullrich M. S., Silvestru C., Kortz U. (2015). Chem. – Eur. J..

[cit63] Yang P., Lin Z., Bassil B. S., Alfaro-Espinoza G., Ullrich M. S., Li M.-X., Silvestru C., Kortz U. (2016). Inorg. Chem..

[cit64] Yang P., Lin Z., Alfaro-Espinoza G., Ullrich M. S., Raţ C. I., Silvestru C., Kortz U. (2016). Inorg. Chem..

[cit65] Liu H., Zou Y.-L., Zhang L., Liu J.-X., Song C.-Y., Chai D.-F., Gao G.-G., Qiu Y.-F. (2014). J. Coord. Chem..

[cit66] Li L., Sha J.-Q., Zong X.-M., Liu C.-J., Zhang Q.-N., Wang D.-W., Yang X.-N., Wang Y. (2014). J. Mol. Struct..

[cit67] Maalaoui A., Hajsalem A., Ratel-Ramond N., Akriche S. (2014). J. Cluster Sci..

[cit68] De Jong W. H., Borm P. J. (2008). Int. J. Nanomed..

[cit69] Feldman D. (2016). J. Macromol. Sci., Part A: Pure Appl. Chem..

[cit70] Watari F., Takashi N., Yokoyama A., Uo M., Akasaka T., Sato Y., Abe S., Totsuka Y., Tohji K. (2009). J. R. Soc., Interface.

[cit71] Shah H. S., Al-Oweini R., Haider A., Kortz U., Iqbal J. (2014). Toxicol. Rep..

[cit72] Draget K. I., Värum K. M., Moen E., Gynnild H., Smidsrød O. (1992). Biomaterials.

[cit73] Meißner T., Bergmann R., Oswald J., Rode K., Stephan H., Richter W., Zänker H., Kraus W., Emmerling F., Reck G. (2006). Transition Met. Chem..

[cit74] Goy R. C., de Britto D., Assis O. B. G. (2009). Polimeros.

[cit75] Feng Y., Han Z., Peng J., Lu J., Xue B., Li L., Ma H., Wang E. (2006). Mater. Lett..

[cit76] Fiorani G., Saoncella O., Kaner P., Altinkaya S. A., Figoli A., Bonchio M., Carraro M. (2014). J. Cluster Sci..

[cit77] De Matteis L., Mitchell S. G., de la Fuente J. M. (2014). J. Mater. Chem. B.

[cit78] T. L. Riss, R. A. Moravec, A. L. Niles, S. Duellman, H. A. Benink, T. J. Worzella and L. Minor, in Assay Guidance Manual, ed. G. S. Sittampalam, N. P. Coussens, K. Brimacombe, A. Grossman, M. Arkin, D. Auld, C. Austin, J. Baell, B. Bejcek, T. D. Y. Chung, J. L. Dahlin, V. Devanaryan, T. L. Foley, M. Glicksman, M. D. Hall, J. V. Hass, J. Inglese, P. W. Iversen, S. D. Kahl, S. C. Kales, M. Lal-Nag, Z. Li, J. McGee, O. McManus, T. Riss, O. J. Trask, J. R. Weidner, M. Xia and X. Xu, Eli Lilly & Company and the National Center for Advancing Translational Sciences, Bethesda (MD), 2004. 22553861

[cit79] Le Ouay B., Stellacci F. (2015). Nano Today.

[cit80] Jung W. K., Koo H. C., Kim K. W., Shin S., Kim S. H., Park Y. H. (2008). Appl. Environ. Microbiol..

[cit81] Gao S., Wu Z., Pan D., Lin Z., Cao R. (2011). Thin Solid Films.

[cit82] Daima H. K., Selvakannan P. R., Kandjani A. E., Shukla R., Bhargava S. K., Bansal V. (2014). Nanoscale.

[cit83] Daima H. K., Selvakannan P. R., Shukla R., Bhargava S. K., Bansal V. (2013). PLoS One.

[cit84] Jenssen H., Hamill P., Hancock R. E. W. (2006). Clin. Microbiol. Rev..

[cit85] Kong Y., Pan L., Peng J., Xue B., Lu J., Dong B. (2007). Mater. Lett..

[cit86] Li J., Chen Z., Zhou M., Jing J., Li W., Wang Y., Wu L., Wang L., Wang Y., Lee M. (2016). Angew. Chem., Int. Ed..

[cit87] Wu K. H., Yu P. Y., Yang C. C., Wang G. P., Chao C. M. (2009). Polym. Degrad. Stab..

[cit88] Yang F.-C., Wu K.-H., Lin W.-P., Hu M.-K. (2009). Microporous Mesoporous Mater..

[cit89] Hu S., Ma C., Zhan F., Cao Y., Hu P., Zhen Q. (2017). Chem. Pap..

[cit90] Chen D., Peng J., Pang H., Zhang P., Chen Y., Chen C., Ma H. (2014). Z. Naturforsch..

[cit91] Ginimuge P. R., Jyothi S. D. (2010). J. Anaesthesiol., Clin. Pharmacol..

[cit92] Yu X., Chen C., Peng J., Shi Z., Shen Y., Mei J., Ren Z. (2014). Thin Solid Films.

[cit93] Herrmann S., De Matteis L., de la Fuente J. M., Mitchell S. G., Streb C. (2017). Angew. Chem., Int. Ed..

[cit94] Kubo A.-L., Kremer L., Herrmann S., Mitchell S. G., Bondarenko O. M., Kahru A., Streb C. (2017). ChemPlusChem.

[cit95] Aureliano M., Ohlin C. A. (2014). J. Inorg. Biochem..

[cit96] Aureliano M. (2016). Oxid. Med. Cell. Longevity.

[cit97] Gândara R. M. C., Soares S. S., Martins H., Gutiérrez-Merino C., Aureliano M. (2005). J. Inorg. Biochem..

[cit98] Soares S. S., Martins H., Duarte R. O., Moura J. J. G., Coucelo J., Gutiérrez-Merino C., Aureliano M. (2007). J. Inorg. Biochem..

[cit99] Tiago D. M., Laizé V., Cancela M. L., Aureliano M. (2008). Cell Biol. Toxicol..

[cit100] Soares S. S., Martins H., Gutiérrez-Merino C., Aureliano M. (2008). Comp. Biochem. Physiol., Part C: Toxicol. Pharmacol..

[cit101] Soares S. S., Gutiérrez-Merino C., Aureliano M. (2007). J. Inorg. Biochem..

[cit102] López X., Bo C., Poblet J. M. (2002). J. Am. Chem. Soc..

[cit103] Ni L., Greenspan P., Gutman R., Kelloes C., Farmer M. A., Boudinot F. D. (1996). Antiviral Res..

[cit104] Keita B., Essaadi K., Nadjo L., Desmadril M. (1995). Chem. Phys. Lett..

[cit105] Sami P., Anand T. D., Premanathan M., Rajasekaran K. (2010). Transition Met. Chem..

[cit106] Seko A., Yamase T., Yamashita K. (2009). J. Inorg. Biochem..

