# Supplementary material for: The antibacterial activity of polyoxometalates: structures, antibiotic effects and future perspectives
Source: Chem Commun (Camb). 2018 Jan 12;54(10):1153–69. doi: 10.1039/c7cc07549a (PMC5804480; doi:10.1039/c7cc07549a)
Supplement: Supplementary file 1 [file CC-054-C7CC07549A-s001.pdf]

Electronic Supplementary Material (ESI) for ChemComm.
This journal is © The Royal Society of Chemistry 2018

Supplementary Information

The antibacterial activity of polyoxometalates: Structures, antibiotic effects and future perspectives

Aleksandar Bijelic,[a] Manuel Aureliano[b] and Annette Rompel[a*]

[a] Universität Wien, Fakultät für Chemie, Institut für Biophysikalische Chemie, Althanstraße 14, 1090 Wien, Austria; www.bpc.univie.ac.at, correspondence to:annette.rompel@univie.ac.at.

[b] CCMar, FCT, Faculdade de Ciências e Tecnologia, Universidade do Algarve, 8000-139 Faro, Portugal.

Content

1. Minimum inhibitory concentration (MIC) Tables 2

2. Figures 7

3. Structure-activity graphs 7

4. References 14

1. Minimum inhibitory concentration (MIC) Tables

Please note that some POMs, which were investigated with regard to their antibacterial activity, are missing in the provided tables due to various reasons but mostly due to missing information in the respective publication. Furthermore, if reported by the authors, the fractional inhibitory concentration (FIC) index is provided for the POMs/POM-hybrids that exhibited/enhanced antibacterial activity in synergy with conventional antibiotics. The FIC index describes the synergy between two drugs based on the MIC of each drug in combination and when used alone.

Table S1 Antibacterial effect of POMs alone (MIC) and in combination with oxacillin (FIC) against MRSA strains SR3605 and ATCC43300.

	**MIC (µg/ml)**		**FIC[a]**		
**POM**	**SR3605[b]**	**ATCC43300[c]**	**SR3605[b]**	**ATCC43300[c]**	**Ref.**
**Polyoxotungstate:**					
**Keggin:**					
$Na_3[PW_{12}O_{40}]$	3200	3200	0.156	0.062	1
$Na_4[SiW_{12}O_{40}]$	3200	3200	0.094	0.019	1
$K_5[BW_{12}O_{40}]$	800	800	0.156	0.094	1
$K_7[PTi_2W_{10}O_{40}]$	12800	3200	0.047	0.063	1,2
$(Pr^{i}NH_3)_6H[PTi_2W_{10}O_{38}(O_2)_2]$	6400	3200	0.094	0.094	2
$(Pr^{i}_2NH_2)_5[PTiW_{11}O_{40}]$	12800	3200	0.039	0.031	2
$(Pr^{i}_2NH_2)_5[PTiW_{11}O_{39}(O_2)]$	3200	1600	0.039	0.023	2
$K_4[GeW_{12}O_{40}]$	1600	800	0.188	0.133	2
$K_6[CoW_{12}O_{40}]$	3200	1600	0.078	0.094	1
$K_6[H_2SiNiW_{11}O_{40}]$	6400	3200	0.078	0.012	2
**Lacunary Keggin:**					
α-$K_7[PW_{11}O_{39}]$	12800	6400	0.063	0.035	1
α-$[SiW_{11}O_{39}]^{8-}$	2000	2000	0.041	0.033	3
α-$[SiW_{11}O_{39}Co]^{6-}$	2000	2000	0.033	0.029	3
B-α-$Na_9[PW_9O_{34}]$	12800	12800	0.047	0.031	1
A-β-$Na_8[PW_9O_{34}]$	12800	6400	0.047	0.047	1
A-β-$Na_9[HSiW_9O_{34}]$	12800	6400	0.094	0.018	2
A-α-$Na_{10}[SiW_9O_{34}]$	12800	12800	0.156	0.010	1
B-α-$Na_9[SbW_9O_{33}]$	3200	1600	0.156	0.156	1
**Double Keggin:**					
$K_9H_5[Ge_2Ti_6W_{18}O_{77}]$	50	10	0.133	0.039	2
$K_2Eu_3H_3[Ge_2Ti_6W_{18}O_{77}]$	50	25	0.141	0.047	
$(TBA)_{7.5}H_{6.5}[Si_2Ti_6W_{18}O_{77}]$	200	100	0.070	0.047	2
$(Me_3NH)_8[Si_2Nb_6W_{18}O_{77}]$	100	25	0.063	0.031	2
**Keggin sandwich:**					
$K_{13}[Eu(SiW_{11}O_{39})_2]$	800	400	0.156	0.012	2
$K_{13}[Gd(SiW_{11}O_{39})_2]$	800	400	0.188	0.020	2
$K_{10}[Zn_4(H_2O)_2(PW_9O_{34})_2]$	400	400	0.078	0.047	2
$K_{10}[Co_4(H_2O)_2(PW_9O_{34})_2]$	800	400	0.016	0.020	2
$K_{12}[Cu_3(PW_9O_{34})_2]$	400	200	0.141	0.070	2
**Wells-Dawson:**					
$K_6[P_2W_{18}O_{62}]$	200	100-200	0.070	0.023	2
$Na_9[P_2Nb_3W_{15}O_{62}]$	800	800	0.141	0.023	2
**Lacunary Wells-Dawson:**					
$Na_{12}[P_2W_{15}O_{56}]$	200	100	0.133	0.039	2
$K_{10}[P_2W_{17}O_{61}]$	200	100	0.141	0.063	2
**Decatungstate:**					
$Na_9[EuW_{10}O_{36}]$	3200	1600	0.188	0.313	1
$K_6[GdW_{10}O_{36}]$	3200	1600	0.313	0.281	1
$Na_8[CeW_{10}O_{36}]$	3200	1600	0.313	0.313	1
**Anderson-Evans:**					
$K_{5.5}H_{1.5}[SbW_6O_{24}]$	12800	12800	0.156	0.063	1
$K_6Na_2[MnW_6O_{24}]$	6400	3200	0.281	0.281	1
$Na_3H_6[CrMo_6O_{24}]$	1600	800	1.03	0.50	2

$Na_3H_6[CoMo_6O_{24}]$	1600	800	1.03	0.50	2
**Other structure:**					
$Na_{27}[NaAs_4W_{40}O_{140}]$	400	400	0.281	0.156	1
$K_{18}[KSb_9W_{21}O_{86}]$	200	400	0.313	0.281	1
$Na_{10}[H_2W_{12}O_{42}]$	51200	25600	0.375	0.094	2
$K_{15}H_3[Pr_3(H_2O)_3(SbW_9O_{33})(W_5O_{18})_3]$	3200	1600	0.156	0.188	2
$K_{15}H_3[Eu_3(H_2O)_3(SbW_9O_{33})(W_5O_{18})_3]$	3200	800	0.156	0.250	2
$K_{15}H_3[Ce_3(H_2O)_3(SbW_9O_{33})(W_5O_{18})_3]$	3200	1600	0.156	0.188	2
$(NH_4)_8[H_2Co_2W_{11}O_{40}]$	1600	800	0.188	0.094	2
$Cs_6[P_2W_5O_{23}]$	3200	800	0.141	0.063	2
**Polyoxovanadotungstate:**					
**Keggin:**					
$K_5[PVW_{11}O_{40}]$	800	400	0.156	0.094	2
$K_4[PVW_{11}O_{40}]$	700-800	400	0.281	0.047	2
$K_5[PV_2W_{10}O_{40}]$	3200	1600	0.156	0.063	2
$K_5[SiVW_{11}O_{40}]$	3200	800	0.125	0.031	1
$K_6[BVW_{11}O_{40}]$	800	200	0.094	0.156	1
$K_7[BVW_{11}O_{40}]$	12800	3200	0.063	0.063	1
$K_6[PV_3W_9O_{40}]$	3200	1600	0.094	0.094	1
$K_6H[SiV_3W_9O_{40}]$	6400	1600	0.125	0.020	2
**Lindqvist:**					
$(MeH_3)_4[VW_5O_{19}]$	1600	800	0.375	0.313	2
**Polyoxomolybdotungstate:**					
**Keggin:**					
$K_3[PMo_3W_9O_{40}]$	3200	1600	0.125	0.094	2
$K_3[PMo_9W_3O_{40}]$	25600	6400	0.500	0.281	2
**Polyoxomolybdate:**					
**Keggin:**					
$(NH_4)_6H[PMo_{11}ZnO_{40}]$	3200	3200	0.501	0.141	2
**Octamolybdate:**					
$Na_2[Mo_8O_{26}(\mathit{L}\text{-lys})_2]$	1600	1600	1.03	1.03	2
$Na_2[Mo_8O_{26}(\mathit{DL}\text{-ala})_2]$	1600	1600	1.03	1.03	2
$Na_2[Mo_8O_{26}(gly)_2]$	1600	1600	1.03	1.03	2
**Other structures:**					
$Na_2[(Hamp)_2Mo_5O_{15}]$	3200	3200	0.50	1.03	2
$Na_6[P_2Mo_5O_{23}]$	6400	6400	0.50	0.188	2
$(NH_4)_6[MnMo_9O_{32}]$	1600	800	1.01	0.75	2
$(NH_4)_{12}H_2[Eu_4(MoO_4)(H_2O)_{16}(Mo_7O_{24})_4]$	>400	>400	<0.562	<0.562	2
$(NH_4)_{12}H_2[Gd_4(MoO_4)(H_2O)_{16}(Mo_7O_{24})_4]$	>400	>400	<0.562	<0.562	2
**Polyoxovanadomolybdate:**					
$Na_6[Mo_6V_2O_{26}]$	3200	1600	1.03	1.00	2
$K_5Na[HMo_9V_3O_{38}]$	3200	1600	1.03	1.00	2
**Polyoxovanadate:**					
**Decavanadate:**					
$(NH_4)_6[V_{10}O_{28}]$	50[d]	-	-	-	4
**Metavanadate:**					
$(TBA)_4[V_4O_{12}]$	8000-16000	8000-16000	-	-	5
**Other structure:**					
$K_7[NiV_{13}O_{38}]$	1000-2000	1000-2000	-	-	5
$K_{10}H_2[V_{18}O_{42}(H_2O)]$	1600	800	1.03	1.03	2
$Na_4H_{15}[V_{12}B_{32}O_{84}]$	800	800	1.00	0.75	2
**Polyoxoniobates/-tantalate:**					
**Lindqvist:**					
$Na_7H[Nb_6O_{19}]$	3200	3200	1.00	0.094	2
$Na_8[Ta_6O_{19}]$	12800	51200	1.00	0.50	2

**Other structure:**					
$Na_7H_{19}[(Eu_3O(OH)_3(OH_2)_3)_2Al_2(Nb_6O_{19})_5]$	800	200	1.50	0.75	2

[a] Fractional inhibitory concentration (FIC) index is defined as: FIC of drug A + FIC of drug B, where FIC of drug A = MIC of drug A in combination / MIC of drug A alone and FIC of drug B = MIC of drug B in combination / MIC of drug B alone. [b] SR3605 is a constitutive methicillin-resistant *S. aureus* (MRSA) strain. [c] ATCC43300 is an inducible methicillin-resistant *S. aureus* (MRSA) strain. [d] The exact MRSA strain, on which the antibacterial activity was tested, was not defined by the authors. amp = adenosine-5'-monophosphate. Pr^iNH_3 = isopropylammonium, $Pr^i_2NH_2$ = diisopropylammonium, TBA = tetra-*n*-butyl ammonium, Me = methyl.

Table S2 Antibacterial activity of POMs alone (MIC) and in combination with β-lactam antibiotics (FIC) against both MSSA strains NCTC8325 and ATCC29213 and antibacterial activity of POMs alone against VRSA strains Mu3 and Mu50.

	MIC (µg/ml)				FIC[a]		
**POM**	**NCTC 8325[b]**	**ATCC 29213[c]**	**Mu3[d]**	**Mu50[d]**	**NCTC 8325[b]**	**ATCC 29213[c]**	**Ref.**
**Polyoxotungstate:**							
**Keggin:**							
$K_7[PTi_2W_{10}O_{40}]$	-	-	76460[e]	76460[e]	-	-	6
**Lacunary Keggin:**							
α-$[PW_{11}O_{39}]^{7-}$	2000	2000	-	-	0.55	0.55	3
α-$[SiW_{11}O_{39}]^{8-}$	2000	2000	-	-	0.53	0.53	3
α-$[SiW_{11}O_{39}Co]^{6-}$	2000	2000	-	-	0.30	0.30	3
**Wells-Dawson:**							
$K_6[P_2W_{18}O_{62}]$	-	-	1940[e]	970[e]	-	-	6
**Polyoxomolybdate:**							
**Keggin:**							
$K_4[SiMo_{12}O_{40}]$	-	-	1625[e]	1625[e]	-	-	6
**Polyoxovanadate:**							
**Metavanadate:**							
$(TBA)_4[V_4O_{12}]$	8000	-	-	-	-	-	5
**Other structure:**							
$K_7[MnV_{13}O_{38}]$	500	-	-	-	-	-	5

[a] Fractional inhibitory concentration (FIC) index is defined as: FIC of drug A + FIC of drug B, where FIC of drug A = MIC of drug A in combination / MIC of drug A alone and FIC of drug B = MIC of drug B in combination / MIC of drug B alone. [b] NCTC8325 is a methicillin-susceptible *S. aureus* (MSSA) strain, which is penicillinase negative. [c] ATCC29213 is a methicillin-susceptible *S. aureus* (MSSA) strain, which is penicillinase positive. [d] Mu3 and Mu50 are a vancomycin-resistant *S. aureus* (VRSA) strain with lowered susceptibility to glycopeptide antibiotics having thicker cell walls in comparison to other resistant strains (In comparison to Mu50, Mu3 has a heterogeneous vancomycin resistance pattern). [e] The MIC values were provided in µM by the authors and therefore had to be converted into µg/ml units by us applying the reported POM formula including crystal waters. TBA = tetra-*n*-butyl ammonium.

Table S3 Antibacterial activity of POMs alone (MIC) against six strains of *S. pneumoniae*.

POM	MIC[a] (μg/ml)	Ref.
**Polyoxotungstate:**		
**Keggin:**		
$Na_3[PW_{12}O_{40}]$	2000-4000	5
$K_7[PTi_2W_{10}O_{40}]$	4000-8000	5
**Decatungstate:**		
$Na_9[EuW_{10}O_{36}]$	2000-4000	5
**Other structure:**		
$K_{18}[KSb_9W_{21}O_{86}]$	128-1000	5
**Polyoxovanadotungstate:**		
**Keggin:**		
$K_5[PVW_{11}O_{40}]$	1000-2000	5
$K_6[BVW_{11}O_{40}]$	1000-4000	5
$K_7[BVW_{11}O_{40}]$	500-2000	5
$K_6[PV_3W_9O_{40}]$	2000-4000	5
**Lindqvist:**		
$(MeH_3N)_4[VW_5O_{19}]$	64-128	5
**Polyoxomolybdate:**		
**Keggin:**		
$Na_3[PMo_{12}O_{40}]$	2000-8000	5
**Octamolybdate:**		
$(Pr^iNH_3)_4[Mo_8O_{26}]$	512-1000	5
**Heptamolybdate:**		
$(NH_4)_6[Mo_7O_{24}]$	512-1000	5
**Polyoxovanadomolybdate:**		
$Na_6[Mo_6V_2O_{26}]$	128-256	5
$K_5Na[HMo_9V_3O_{38}]$	128-256	5
**Polyoxovanadate:**		
**Decavanadate:**		
$(TBA)_4[V_{10}O_{28}]$	4-8	5
**Metavanadate:**		
$(TBA)_4[V_4O_{12}]$	8-32	5
**Other structure:**		
$K_7[MnV_{13}O_{38}]$	8-32	5
$K_7[NiV_{13}O_{38}]$	8-32	5
$K_5H_2[V_{15}O_{36}(CO_3)]$	4-16	5
$K_{10}H_2[V_{18}O_{42}(H_2O)]$	8-16	5

[a] Six *S. pneumoniae* strains were tested, namely penicillin-intermediate-resistant IID553 and IID554 and penicillin-resistant BS225, BS234, BS259 and BS269. Pr^iNH_3 = isopropylammonium, TBA = tetra-*n*-butyl ammonium , Me = methyl.

Table S4 Antibacterial activity of POM-hybrids and nanocomposites against a series of bacterial strains.

	MIC (µg/ml)								
**POM/POM-hybrid**	**SA[a]**	**EC[b]**	**BS[c]**	**PA[d]**	**Psp[e]**	**Vsp[f]**	**PP[g]**	**CM[h]**	**Ref.**
**Polyoxovanadate:**									
**Decavanadate:**									
$(NH_4)_6[V_{10}O_{28}]$	50	50	-	-	-	-	-	-	4
**Organic-inorganic-POM:**									
**Organoantimony-polyoxotungstate:**									
$(NH_4)_{12}[(PhSb^{III})_4(A\text{-}\alpha\text{-}Ge^{IV}W_9O_{34})_2]$	-	80	40-80	-	-	-	-	-	7
$Rb_9Na[(PhSb^{III})_4(A\text{-}\alpha\text{-}P^{V}W_9O_{34})_2]$	-	110	50	-	-	-	-	-	7
$Rb_3[\{2\text{-}(Me_2NCH_2C_6H_4)Sb^{III}\}_3(B\text{-}\alpha\text{-}As^{III}W_9O_{33})]$	-	130	60	-	-	-	-	-	7
$Cs_3K_{3.5}Na_{4.5}[(PhSb^{III})\{Na(H_2O)\}As^{III}{}_2W_{19}O_{67}(H_2O)]$	-	500	125	-	250	125	125	250	8
$Cs_{4.5}K_{5.5}[(PhSb^{III})_2As^{III}{}_2W_{19}O_{67}(H_2O)]$	-	250	62.5	-	125	62.5	62.5	250	8
$Cs_{4.5}Na_{7.5}[(PhSb^{III})_3(B\text{-}\alpha\text{-}As^{III}W_9O_{33})_2]$	-	125	62.5	-	62.5	31.3	15.6	<7.8	8
$Cs_{6.5}Na_{3.5}[(PhSb^{III})_4(A\text{-}\alpha\text{-}As^{V}W_9O_{34})_2]$	-	62.5	15.6	-	15.6	15.6	<7.8	<7.8	9
$Rb_{9.25}Na_{0.75}[(OHSb^{III})_4(A\text{-}\alpha\text{-}As^{V}W_9O_{34})_2]$	-	1000	250	-	1000	1000	1000	125	9
$Cs_3KNa_6[Na\{2\text{-}(Me_2HN^+CH_2)C_6H_4Sb^{III}\}As^{III}{}_2W_{19}O_{67}(H_2O)]$	-	1000	250	-	500	250	500	500	10
$Rb_{2.5}K_{5.5}[\{2\text{-}(Me_2HN^+CH_2)C_6H_4Sb^{III}\}_2As^{III}{}_2W_{19}O_{67}(H_2O)]$	-	500	250	-	250	250	1000	250	10
**Quinolone-based drug-POM:**									
$[Co^{II}(C_{19}FH_{22}N_3O_4)_3][C_{19}FH_{23}N_3O_4][HSiW_{12}O_{40}]$	2.52	2.42	-	-	-	-	-	-	11
**Nanocomposite:**									
**Bamboo charcoal-POM:**									
BC/POM[i]	4	4	4	4	-	-	-	-	12
**Polymer-POM:**									
**PVA/PEI-POM:**									
$PVA\text{-}PEI\text{-}H_5PV_2Mo_{10}O_{40}$[i]	0.02	2	0.2	0.2	-	-	-	-	13
**Chitosan-POM:**									
$CTS\text{-}Ca_3V_{10}O_{28}$	12.5	12.5	-	-	-	-	-	-	4
**Polyoxometalate ionic liquids:**									
$[N(C_6H_{13})_4]_8[\alpha\text{-}SiW_{11}O_{39}]$	10	1000	-	1000	-	-	-	-	14
$[N(C_7H_{15})_4]_8[\alpha\text{-}SiW_{11}O_{39}]$	2	25	-	100	-	-	-	-	14
$[N(C8H_{17})_4]_8[\alpha\text{-}SiW_{11}O_{39}]$	5	50	-	100	-	-	-	-	14

[a] *S. aureus* (no further information about the exact strain). [b] *E. coli* (partially the JM109 strain was used, which is resistant towards nalidixic acid but provided similar/same results as the DH5α strain). [c] *B. subtilis* (no further information about the exact strain). [d] *P. aeruginosa* (no further information about the exact strain). [e] Paenibacillus sp. (no further information about the exact strain). [f] *Vibrio sp.* Gal12. [g] *P. putida* DSM291. [h] *C. michiganensis* (no further information about the exact strain). [i] A series of these nanocomposites differing in POM concentration (within the matrix) were tested and only the result of the highest POM concentration is shown (BC/POM ratio = 1:3 and PVA-PEI/POM ratio = 1:10).

2. Figures

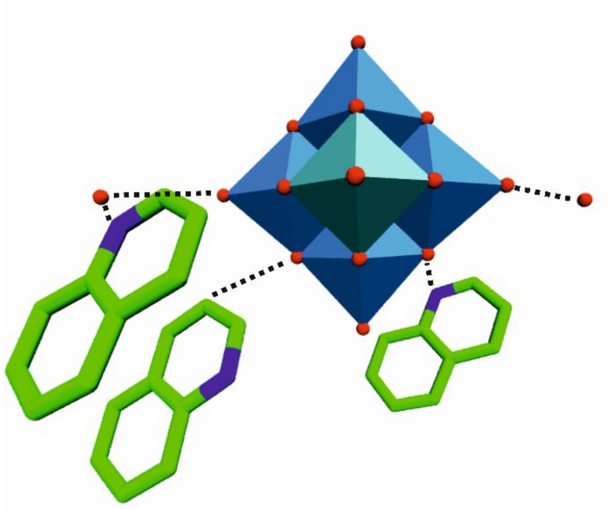

Fig. S1 Structure of $(C_9H_8N)_3[NbW_5O_{19}]$. Structure of the organic-inorganic hybrid is shown, with the POM $[NbW_5O_{19}]^{3-}$ being represented as polyhedra and the organic quinolinium cations as ball and stick. Color code: Tungsten, blue; niobium, cyan; carbon, green; nitrogen, dark blue; oxygen, red. Dashed lines indicate hydrogen-bonds between the inorganic and organic entities.

3. Structure-activity graphs

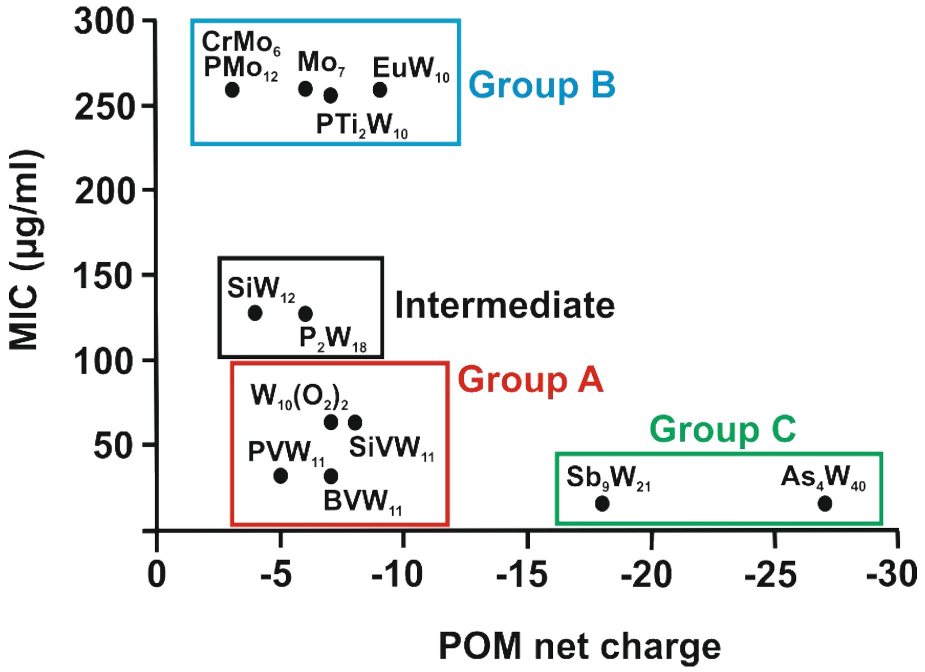

Fig. S2 Charge-activity-relationship of POMs against the 10 drug susceptible strains of *Helicobacter pylori*. The net charge of the POMs is plotted against their MIC values. Specific groups are marked, namely group A (red) of POMs with higher activity (MIC up to 100 µg/ml), group B (blue) of POMs with lower activity (MIC > 200 µg/ml) and group C (green) of large and highly charged POMs exhibiting the highest activity. Please note that the MIC values are average values originating from in total 10 tested drug susceptible strains. Between MIC values of 100 and 200 µg/ml there is an intermediate zone (black) representing moderately active POMs. $CrMo_6$ = $[CrMo_6O_{24}H_6]^{3-}$, PMo_{12} = $[PMo_{12}O_{40}]^{3-}$, Mo_7 = $[Mo_7O_{24}]^{6-}$, EuW_{10} = $[EuW_{10}O_{36}]^{9-}$, P_2W_{18} = $[P_2W_{18}O_{62}]^{6-}$, PVW_{11} = $[PVW_{11}O_{40}]^{5-}$, PTi_2W_{10} = $[PTi_2W_{10}O_{40}]^{7-}$, $W_{10}(O_2)_2$ = $[PTi_2W_{10}O_{38}(O_2)_2]^{7-}$, SiW_{12} = $[SiW_{12}O_{40}]^{4-}$, $SiVW_{11}$ = $[SiVW_{11}O_{40}]^{7-}$, BVW_{11} = $[BVW_{11}O_{40}]^{7-}$, Sb_9W_{21} = $[KSb_9W_{21}O_{86}]^{18-}$, As_4W_{40} = $[KAs_4W_{40}O_{140}]^{27-}$.

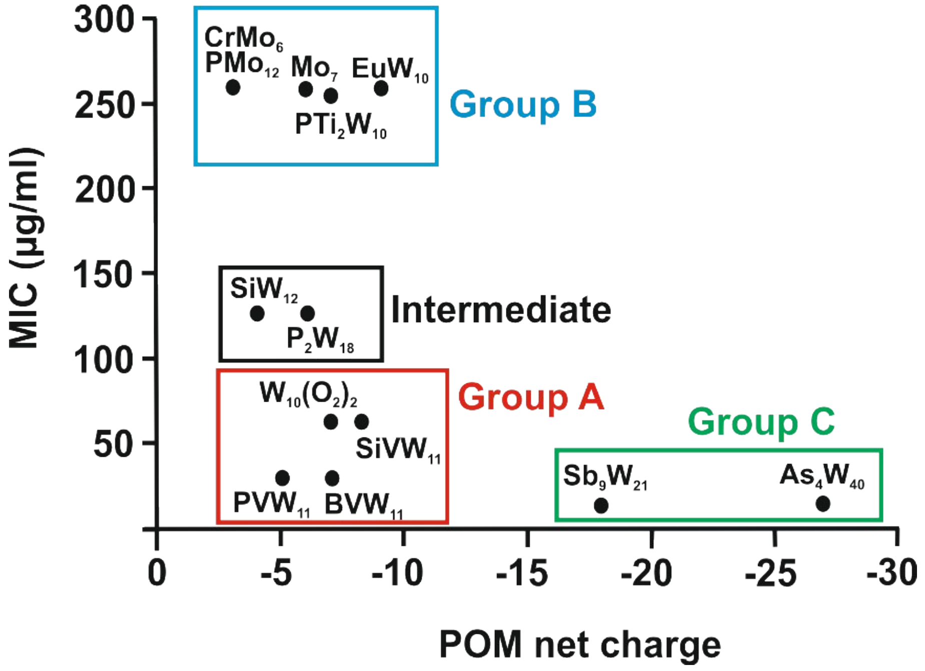

Fig. S3 Charge-activity-relationship of POMs against the metronidazole-resistant strain Hp018 of *Helicobacter pylori*. The net charge of the POMs is plotted against their MIC values. Specific groups are marked, namely group A (red) of POMs with higher activity (MIC up to 100 µg/ml), group B (blue) of POMs with lower activity (MIC > 200 µg/ml) and group C (green) of large and highly charged POMs exhibiting the highest activity. Between MIC values of 100 and 200 µg/ml there is an intermediate zone (black) representing moderately active POMs. For full POM formula, see caption of Figure S2.

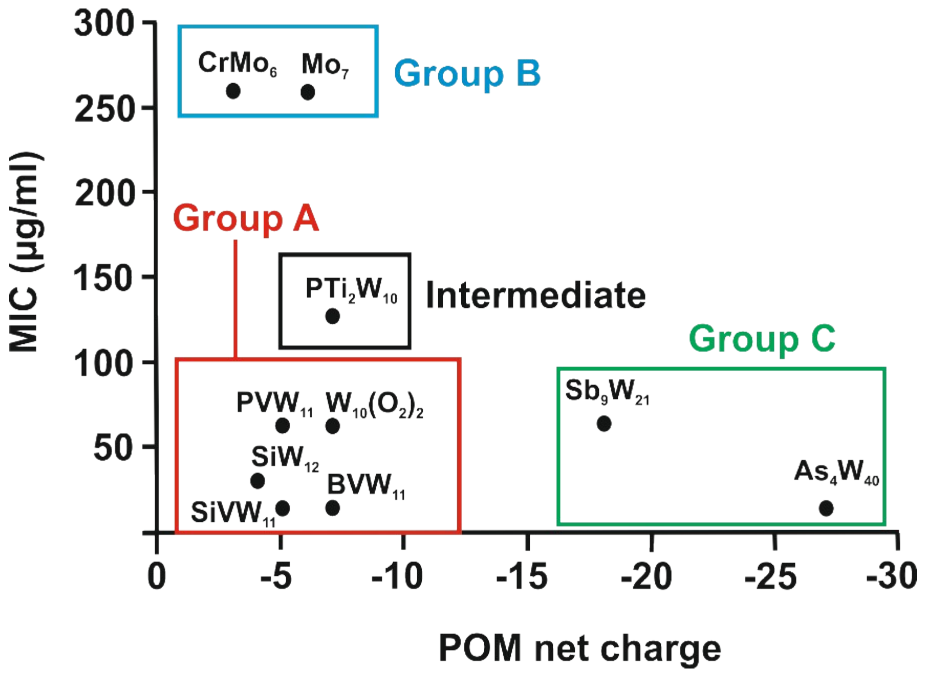

Fig. S4 Charge-activity-relationship of POMs against the metronidazole-resistant strain Hp030 of *Helicobacter pylori*. The net charge of the POMs is plotted against their MIC values. Specific groups are marked, namely group A (red) of POMs with higher activity (MIC up to 100 µg/ml), group B (blue) of POMs with lower activity (MIC > 200 µg/ml) and group C (green) of large and highly charged POMs exhibiting the highest activity. Between MIC values of 100 and 200 µg/ml there is an intermediate zone (black) representing moderately active POMs. Note that this is the only tested strain, where a group C member (Sb_9W_{21}) exhibits a MIC value > 50 µg/ml. In addition, no data are available for the POMs PMo_{12}, EuW_{10} and P_2W_{18} against this strain. For full POM formula, see caption of Figure S2.

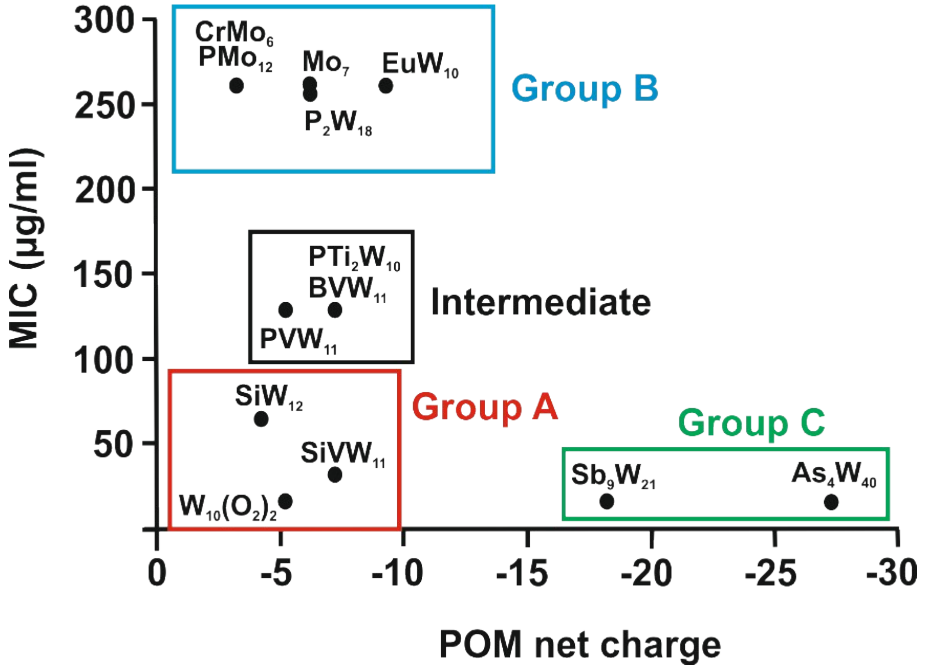

Fig. S5 Charge-activity-relationship of POMs against the metronidazole-resistant strain Hp065 of *Helicobacter pylori*. The net charge of the POMs is plotted against their MIC values. Specific groups are marked, namely group A (red) of POMs with higher activity (MIC up to 100 µg/ml), group B (blue) of POMs with lower activity (MIC > 200 µg/ml) and group C (green) of large and highly charged POMs exhibiting the highest activity. Between MIC values of 100 and 200 µg/ml there is an intermediate zone (black) representing moderately active POMs. For full POM formula, see caption of Figure S2.

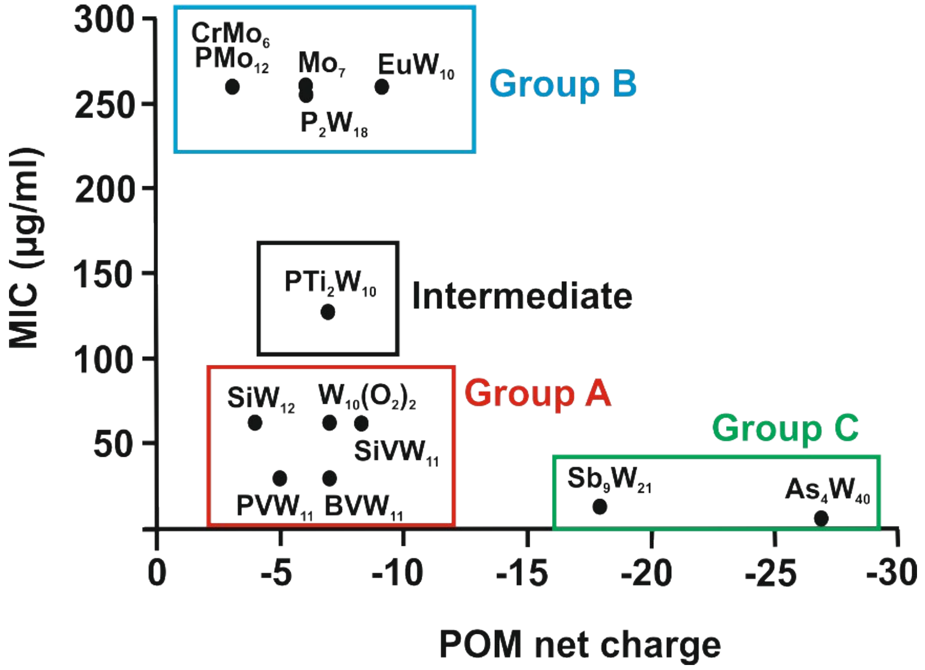

Fig. S6 Charge-activity-relationship of POMs against the metronidazole-resistant strain ATCC43504 of *Helicobacter pylori*. The net charge of the POMs is plotted against their MIC values. Specific groups are marked, namely group A (red) of POMs with higher activity (MIC up to 100 µg/ml), group B (blue) of POMs with lower activity (MIC > 200 µg/ml) and group C (green) of large and highly charged POMs exhibiting the highest activity. Between MIC values of 100 and 200 µg/ml there is an intermediate zone (black) representing moderately active POMs. For full POM formula, see caption of Figure S2.

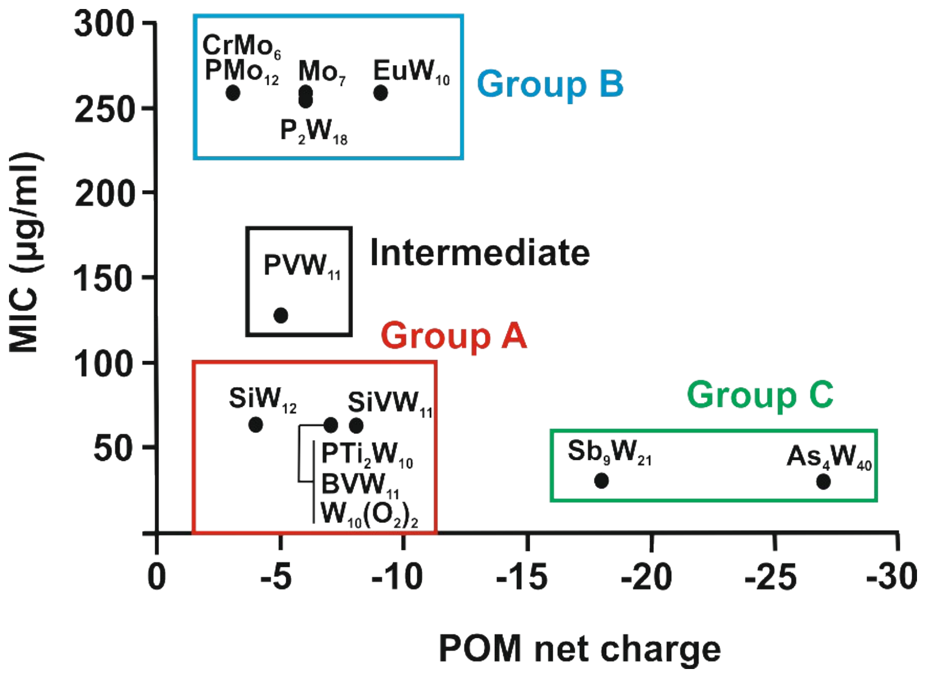

Fig. S7 Charge-activity-relationship of POMs against the clarithromycin-resistant strain Hp067 of *Helicobacter pylori*. The net charge of the POMs is plotted against their MIC values. Specific groups are marked, namely group A (red) of POMs with higher activity (MIC up to 100 µg/ml), group B (blue) of POMs with lower activity (MIC > 200 µg/ml) and group C (green) of large and highly charged POMs exhibiting the highest activity. Between MIC values of 100 and 200 µg/ml there is an intermediate zone (black) representing moderately active POMs. For full POM formula, see caption of Figure S2.

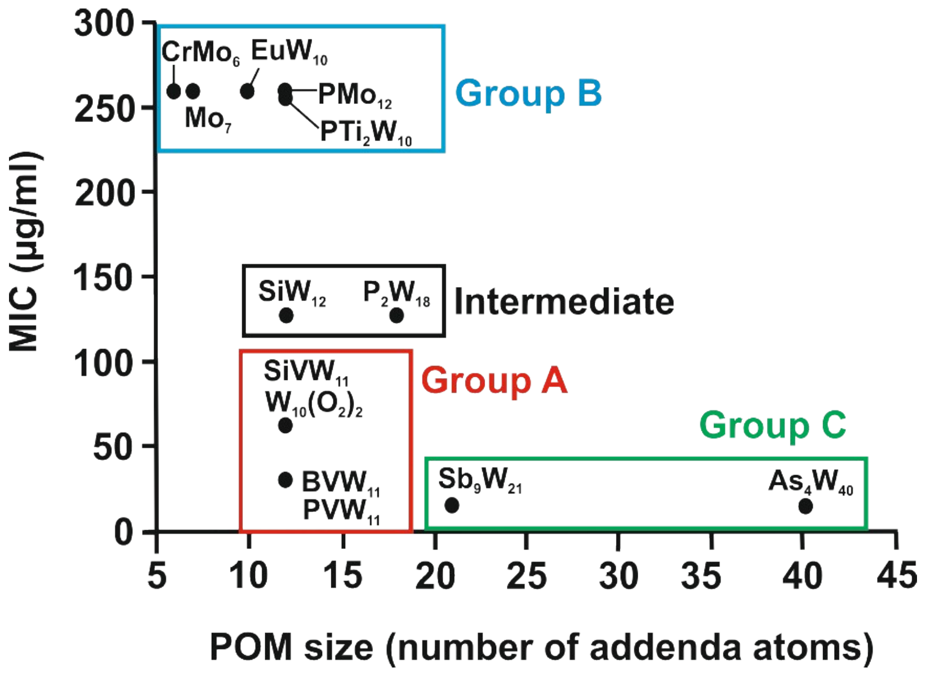

Fig. S8 Size-activity-relationship of POMs against the 10 drug susceptible strains of *Helicobacter pylori*. The net charge of the POMs expressed as number of addenda atoms is plotted against their MIC values. Specific groups are marked, namely group A (red) of POMs with higher activity (MIC up to 100 µg/ml), group B (blue) of POMs with lower activity (MIC > 200 µg/ml) and group C (green) of large and highly charged POMs exhibiting the highest activity. Between MIC values of 100 and 200 µg/ml there is an intermediate zone (black) representing moderately active POMs. For full POM formula, see caption of Figure S2.

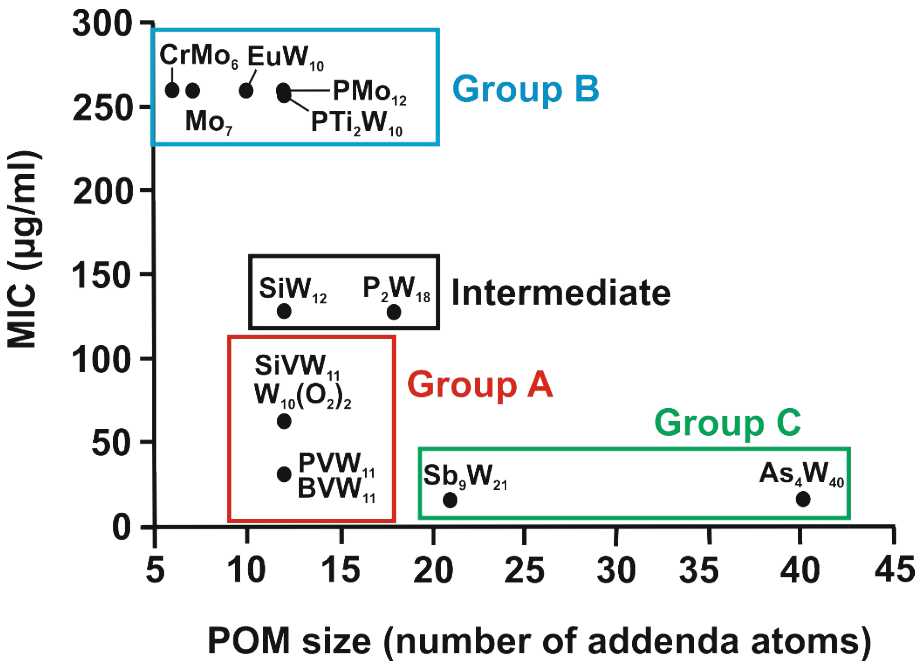

Fig. S9 Size-activity-relationship of POMs against the metronidazole-resistant strain Hp018 of *Helicobacter pylori.* The net charge of the POMs expressed as number of addenda atoms is plotted against their MIC values. Specific groups are marked, namely group A (red) of POMs with higher activity (MIC up to 100 µg/ml), group B (blue) of POMs with lower activity (MIC > 200 µg/ml) and group C (green) of large and highly charged POMs exhibiting the highest activity. Between MIC values of 100 and 200 µg/ml there is an intermediate zone (black) representing moderately active POMs. For full POM formula, see caption of Figure S2.

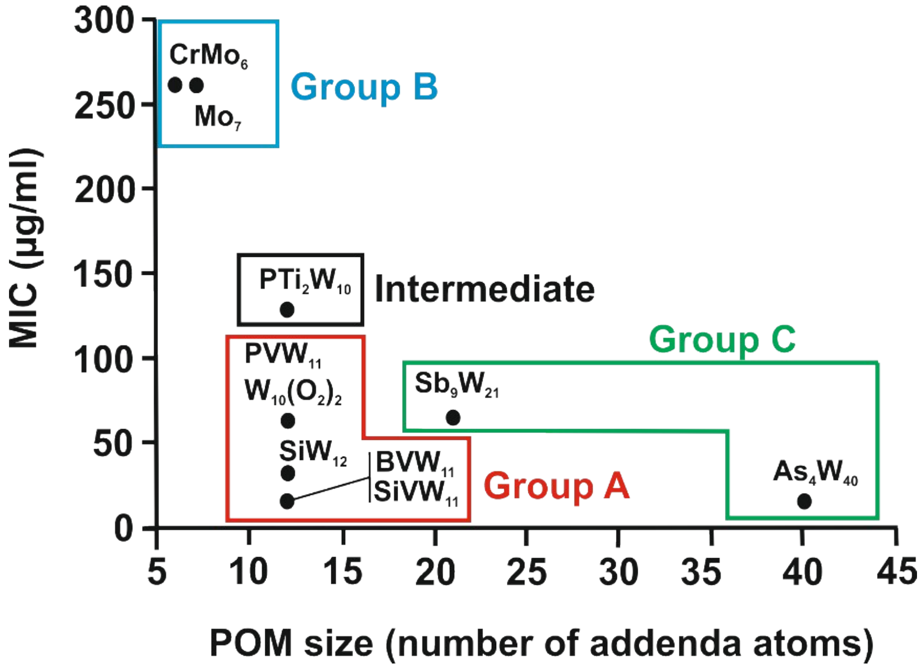

Fig. S10 Size-activity-relationship of POMs against the metronidazole-resistant strain Hp030 of *Helicobacter pylori*. The net charge of the POMs expressed as number of addenda atoms is plotted against their MIC values. Specific groups are marked, namely group A (red) of POMs with higher activity (MIC up to 100 µg/ml), group B (blue) of POMs with lower activity (MIC > 200 µg/ml) and group C (green) of large and highly charged POMs exhibiting the highest activity. Between MIC values of 100 and 200 µg/ml there is an intermediate zone (black) representing moderately active POMs. Note that this is the only tested strain, where a group C member (Sb_9W_{21}) exhibits a MIC value > 50 µg/ml. In addition, no data are available for the POMs PMo_{12}, EuW_{10} and P_2W_{18} against this strain. For full POM formula, see caption of Figure S2.

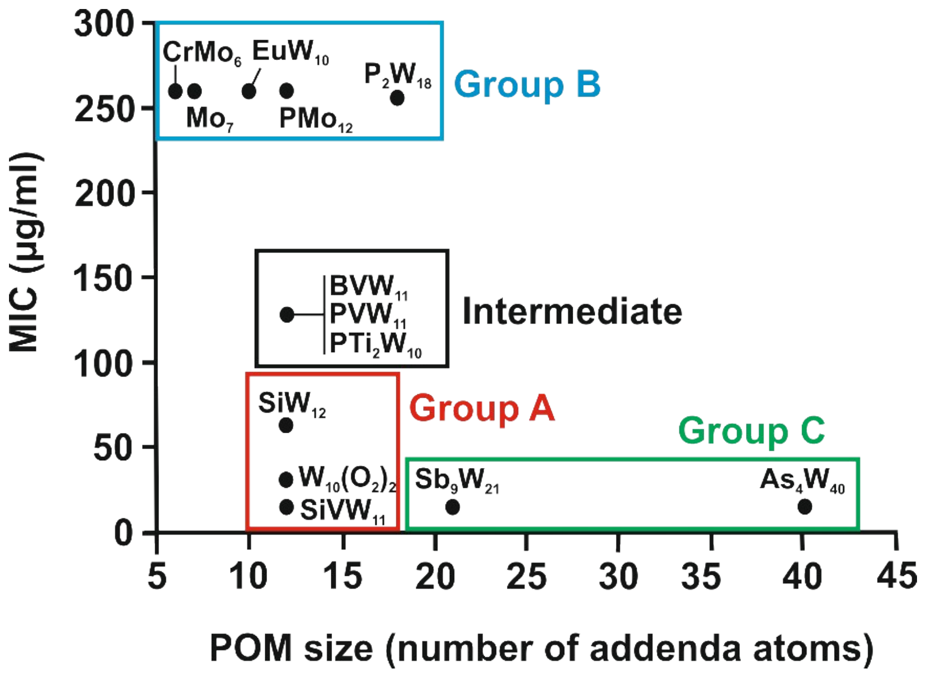

Fig. S11 Size-activity-relationship of POMs against the metronidazole-resistant strain Hp065 of *Helicobacter pylori*. The net charge of the POMs expressed as number of addenda atoms is plotted against their MIC values. Specific groups are marked, namely group A (red) of POMs with higher activity (MIC up to 100 µg/ml), group B (blue) of POMs with lower activity (MIC > 200 µg/ml) and group C (green) of large and highly charged POMs exhibiting the highest activity. Between MIC values of 100 and 200 µg/ml there is an intermediate zone (black) representing moderately active POMs. For full POM formula, see caption of Figure S2.

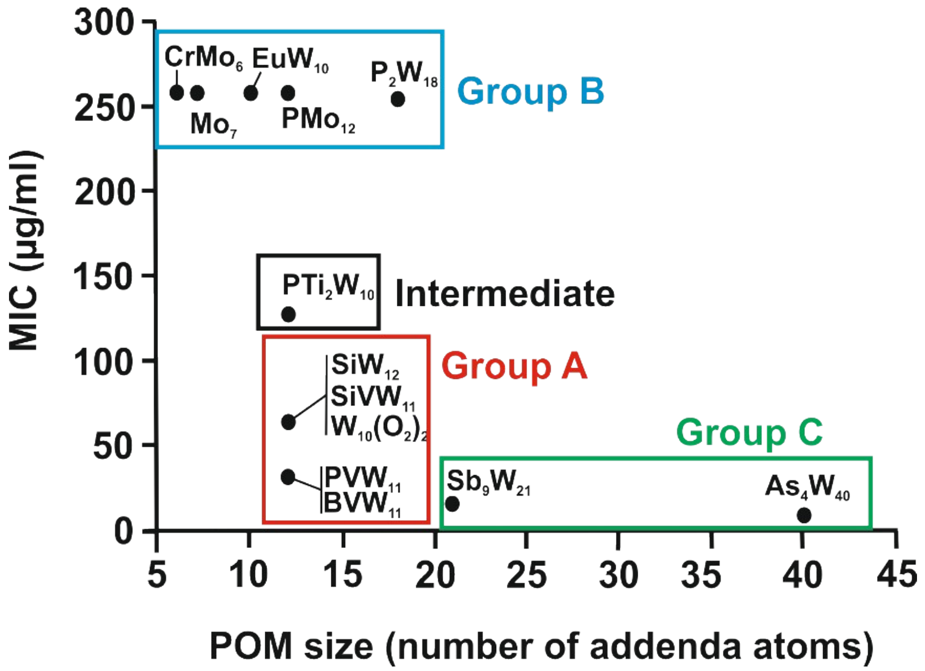

Fig. S12 Size-activity-relationship of POMs against the metronidazole-resistant strain ATCC43504 of *Helicobacter pylori*. The net charge of the POMs expressed as number of addenda atoms is plotted against their MIC values. Specific groups are marked, namely group A (red) of POMs with higher activity (MIC up to 100 µg/ml), group B (blue) of POMs with lower activity (MIC > 200 µg/ml) and group C (green) of large and highly charged POMs exhibiting the highest activity. Between MIC values of 100 and 200 µg/ml there is an intermediate zone (black) representing moderately active POMs. For full POM formula, see caption of Figure S2.

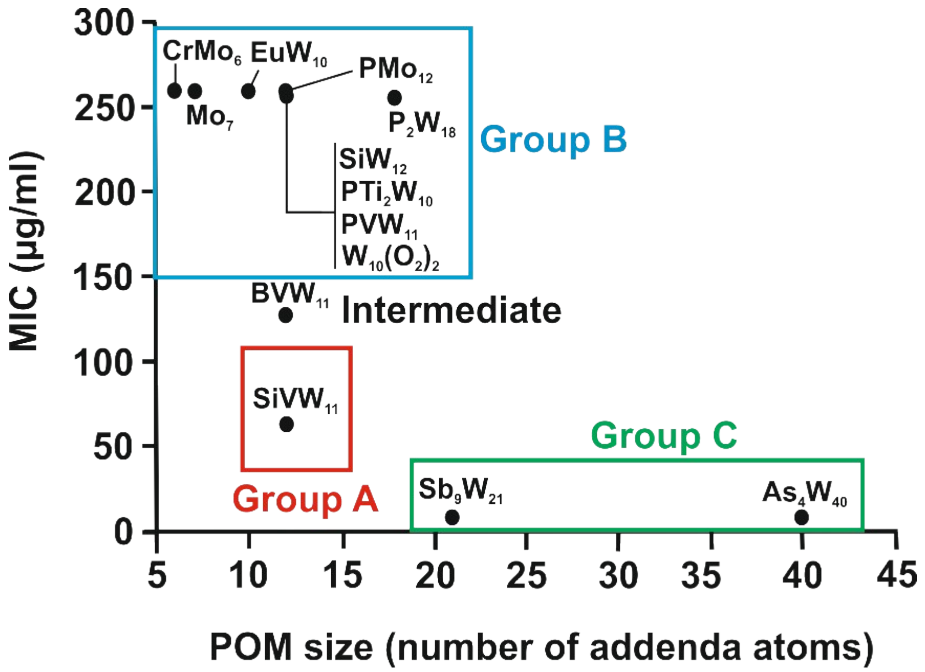

Fig. S13 Size-activity-relationship of POMs against the clarithromycin-resistant strain Hp027 of *Helicobacter pylori*. The net charge of the POMs expressed as number of addenda atoms is plotted against their MIC values. Specific groups are marked, namely group A (red) of POMs with higher activity (MIC up to 100 µg/ml), group B (blue) of POMs with lower activity (MIC > 200 µg/ml) and group C (green) of large and highly charged POMs exhibiting the highest activity. Between MIC values of 100 and 200 µg/ml there is an intermediate zone representing moderately active POMs (i.e. BVW_{11}). For full POM formula, see caption of Figure S2.

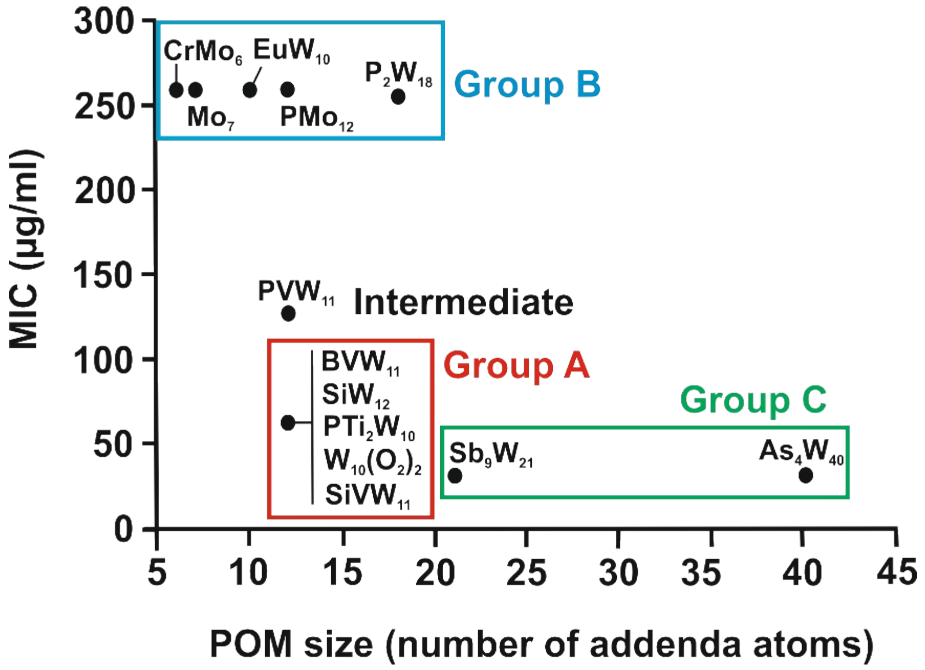

Fig. S14 Size-activity-relationship of POMs against the clarithromycin-resistant strain Hp067 of *Helicobacter pylori*. The net charge of the POMs expressed as number of addenda atoms is plotted against their MIC values. Specific groups are marked, namely group A (red) of POMs with higher activity (MIC up to 100 µg/ml), group B (blue) of POMs with lower activity (MIC > 200 µg/ml) and group C (green) of large and highly charged POMs exhibiting the highest activity. Between MIC values of 100 and 200 µg/ml there is an intermediate zone representing moderately active POMs (i.e. PVW_{11}). For full POM formula, see caption of Figure S2.

4. References

1 T. Yamase, N. Fukuda and Y. Tajima, *Biol. Pharm. Bull.*, 1996, **19**, 459–465.

2 N. Fukuda, T. Yamase and Y. Tajima, *Biol. Pharm. Bull.*, 1999, **22**, 463–470.

3 Y. Tajima, *Biomed. Res.*, 2002, **23**, 115–125.

4 S. Chen, G. Wu, D. Long and Y. Liu, *Carbohydr. Polym.*, 2006, **64**, 92–97.

5 N. Fukuda and T. Yamase, *Biol. Pharm. Bull.*, 1997, **20**, 927–930.

6 M. Inoue, T. Suzuki, Y. Fujita, M. Oda, N. Matsumoto and T. Yamase, *J. Inorg. Biochem.*, 2006, **100**, 1225–1233.

7 M. Inoue, K. Segawa, S. Matsunaga, N. Matsumoto, M. Oda and T. Yamase, *J. Inorg. Biochem.*, 2005, **99**, 1023–1031.

8 M. Barsukova-Stuckart, L. F. Piedra-Garza, B. Gautam, G. Alfaro-Espinoza, N. V. Izarova, A. Banerjee, B. S. Bassil, M. S. Ullrich, H. J. Breunig, C. Silvestru and U. Kortz, *Inorg. Chem.*, 2012, **51**, 12015–12022.

9 P. Yang, B. S. Bassil, Z. Lin, A. Haider, G. Alfaro-Espinoza, M. S. Ullrich, C. Silvestru and U. Kortz, *Chem. – Eur. J.*, 2015, **21**, 15600–15606.

10 P. Yang, Z. Lin, B. S. Bassil, G. Alfaro-Espinoza, M. S. Ullrich, M.-X. Li, C. Silvestru and U. Kortz, *Inorg. Chem.*, 2016, **55**, 3718–3720.

11 P. Yang, Z. Lin, G. Alfaro-Espinoza, M. S. Ullrich, C. I. Raţ, C. Silvestru and U. Kortz, *Inorg. Chem.*, 2016, **55**, 251–258.

12 H. Liu, Y.-L. Zou, L. Zhang, J.-X. Liu, C.-Y. Song, D.-F. Chai, G.-G. Gao and Y.-F. Qiu, *J. Coord. Chem.*, 2014, **67**, 2257–2270.

13 F.-C. Yang, K.-H. Wu, W.-P. Lin and M.-K. Hu, *Microporous Mesoporous Mater.*, 2009, **118**, 467–472.

14 K.-H. Wu, P. Y. Yu, C. C. Yang, G. P. Wang and C. M. Chao, *Polym. Degrad. Stab.*, 2009, **94**, 1411–1418.

15 A.-L. Kubo, L. Kremer, S. Herrmann, S. G. Mitchell, O. M. Bondarenko, A. Kahru and C. Streb, *ChemPlusChem*, 2017, **82**, 867–871.
